# Chemical proteomics reveals target selectivity of clinical Jak inhibitors in human primary cells

**DOI:** 10.1038/s41598-019-50335-5

**Published:** 2019-10-02

**Authors:** H. Christian Eberl, Thilo Werner, Friedrich B. Reinhard, Stephanie Lehmann, Douglas Thomson, Peiling Chen, Cunyu Zhang, Christina Rau, Marcel Muelbaier, Gerard Drewes, David Drewry, Marcus Bantscheff

**Affiliations:** 1 Cellzome GmbH, A GlaxoSmithKline Company, Meyerhofstraße 1, 69117 Heidelberg, Germany; 2 GlaxoSmithKline, Upper Merion, 709 Swedeland Rd #1539, King of Prussia, PA 19406 United States; 3 GlaxoSmithKline, Research Triangle Park, 5 Moore Drive, North Carolina, 27709 United States; 4 Present Address: UNC Eshelman School of Pharmacy, Structural Genomics Consortium, University of North Carolina at Chapel Hill, 120 Mason Farm Rd, Chapel Hill, NC 27599 United States

**Keywords:** Kinases, Proteomics, Target identification

## Abstract

Kinobeads are a set of promiscuous kinase inhibitors immobilized on sepharose beads for the comprehensive enrichment of endogenously expressed protein kinases from cell lines and tissues. These beads enable chemoproteomics profiling of kinase inhibitors of interest in dose-dependent competition studies in combination with quantitative mass spectrometry. We present improved bead matrices that capture more than 350 protein kinases and 15 lipid kinases from human cell lysates, respectively. A multiplexing strategy is suggested that enables determination of apparent dissociation constants in a single mass spectrometry experiment. Miniaturization of the procedure enabled determining the target selectivity of the clinical BCR-ABL inhibitor dasatinib in peripheral blood mononuclear cell (PBMC) lysates from individual donors. Profiling of a set of Jak kinase inhibitors revealed kinase off-targets from nearly all kinase families underpinning the need to profile kinase inhibitors against the kinome. Potently bound off-targets of clinical inhibitors suggest polypharmacology, e.g. through MRCK alpha and beta, which bind to decernotinib with nanomolar affinity.

## Introduction

Kinases are among the most prominent drug targets due to their central role in cellular signalling processes, inflammation and their frequent dysregulation in cancer^1^. As of 2017, the FDA has approved 37 small molecule kinase inhibitors^2^. Kinase inhibitors typically target the ATP binding site which is a well-understood and tractable binding pocket, but the high conservation of the ATP binding site within the kinase family poses a serious challenge to develop highly specific kinase inhibitors^3^. Candidate kinase inhibitors for probe development and drug discovery are usually tested against a panel of recombinant kinases to understand selectivity to other kinases. However, these assays are hampered by the fact that recombinant proteins do not necessarily mimic endogenous full-length proteins, which can be post-translationally modified and incorporated into protein complexes. Unexpected kinase inhibitor off-targets might on the one hand offer opportunities for repurposing. For instance, the finding that Imatinib inhibits cKIT in addition to the primary target BCR-ABL enabled treatment of gastrointernal stroma tumours^4^. On the other hand, kinase off-target findings could explain adverse effects. The cardiotoxicity of sunitinib for example could be linked to the inhibition of AMP-activated protein kinase^5^.

Chemical proteomics approaches such as activity or affinity-based profiling allow assessing selectivity as well as affinity of small molecule inhibitors^6,7^ against their endogenously expressed targets in cell extracts and live cells^8,9^. In a typical experiment, a probe compound is immobilized on a solid support and target proteins are enriched by incubation with a cell lysate. Competitive binding studies with excess free inhibitor enable the identification of specific targets and the binding strength can be determined in dose-dependent competition experiments measuring the reduction of target binding to the bead matrix as a function of free inhibitor concentration. Quantitative mass spectrometric analysis using isobaric mass tags^10^ allows assessing free inhibitor binding to all specifically captured proteins in a single experiment. Probe compounds with a broad inhibition profile within a target class or combinations of unspecific probes can be used to enrich a large fraction of a protein family thus generating a generic binding assay for this target class. For kinases, the combination of several immobilized unselective kinase inhibitors, ‘kinobeads’has enabled a a powerful chemoproteomics approach to monitor the selectivity of kinase inhibitors of interest against a significant fraction of the kinome expressed in cell lines and tissues^11–15^. More than 200 kinases are accessible with current methodologies and recent progress towards miniaturization of kinase protocols suggests application of such profiling approaches to rare cell types and very small tissue amounts^15,16^ but in particular dose dependent experiments with scarce cell material remain challenging.

The Jak/Stat pathway plays a central role in immunity with more than 50 cytokines eliciting their signal via this axis. Central players are the Janus kinases (JAKs) - JAK1, JAK2, JAK3 and TYK2 - which associate with receptors upon cytokine binding. JAK1, JAK2 and TYK2 are ubiquitously expressed, whereas JAK3 is primarily expressed in hematopoietic cells^17^. Receptor binding leads to kinase activation and phosphorylation of the kinase, the cytoplasmic tail of the receptors and the transcription activating Stat proteins^18^. Dysregulation of this pathway is implicated in numerous diseases, including autoimmune diseases, inflammatory diseases and cancer^19–22^. The central enzymatic function in this pathway – the JAK kinases – are the main target for pharmacological inhibition. By 2018, three small molecule inhibitors, Incyte’s roxulitinib (Jakafi), Pfizer’s tofacitinib (Xeljanz) and Ely Lilly’s baricitinib (Olumiant) have achieved FDA approval. The JAK1/JAK2 inhibitor ruxolutinib has been approved for treatment of Myelofibrosis^23^ and polycythemia vera^24^, whereas the pan-JAK inhibitors tofacitinib and baricitinib have been approved for the treatment of rheumatoid arthritis^24–26^. Several further molecules are currently in late stage clinical trials and might reach the market soon^27^.

The importance of kinase inhibitors in drug discovery with more and more applications beyond cancer therapy requires the full characterization of kinase selectivity and safety hazards in disease- or safety-relevant primary material. Here, we set out to optimize kinome coverage and sensitivity of the kinobeads chemoproteomics profiling approach to enable the determination of kinase inhibitor selectivity in scarce primary and in patient-derived samples. We present improved kinobeads matrices which enable the unprecedented coverage of human protein and lipid kinases and demonstrate the application for kinase profiling in preclinical model species as well as parasites. Due to the high specificity of this bead matrix in conjunction with recent technological developments in proteomics, we were able to miniaturize the affinity enrichment procedure allowing work with lysates derived from human peripheral blood mononuclear cells (PBMCs). We then applied this miniaturized kinobeads workflow to profile 11 JAK family inhibitors including tool, clinical and marketed compounds in human PBMC lysate and elucidate their target specificity within the Jak family as well as across the kinome.

## Results

### Establishment of an improved kinase capturing matrix

**Figure 1:**
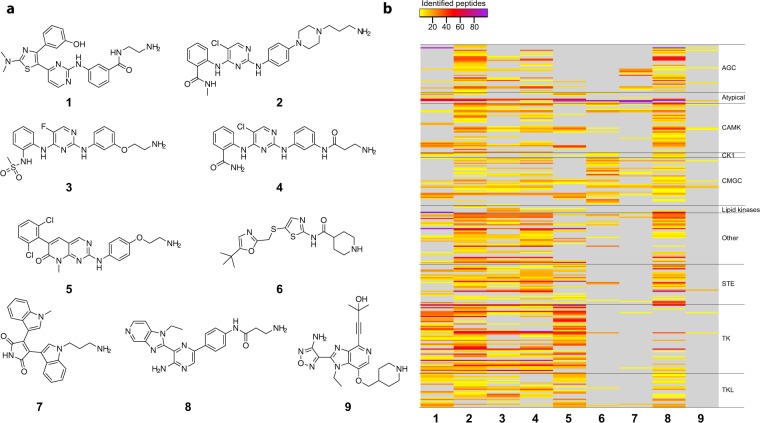
Kinase capturing matrix. (**a**) Chemical structures of the nine kinase capturing compounds. Amines used for coupling are oriented to the right (**b**) Heatmap displaying the number of unique identified peptides for each identified kinase using each individual capturing compound alone. Kinases were categorized according to their subfamilies. Pulldowns were performed using a lysate mix of K562, HEK293 and human placenta. The number of identified peptides was used as a proxy to estimate capturing efficacy (see also Supplemental Table 1).

To improve the kinome coverage of the kinase-capturing matrix and especially to cover kinases like PKA and AKT which previously were challenging targets^11^ we tested a set of promiscuous inhibitors. We generated a library of 55 amine-functionalized kinase inhibitors based on known broad range ATP-competitive kinase inhibitors and kinase inhibitor scaffolds. Each compound was immobilized on beads and tested in a pull-down experiment with a mixture of extracts from HEK293 cells, K562 cells and human placenta to assess the captured part of the kinome. The components of the lysate mix were chosen based on kinase expression (proteomicsDB^28^) and availability. For example, human placenta is a readily available human tissue and contains epithelial and vascular cells that express receptor tyrosine kinases such as FLT4 and KDR (Supplementary Fig. 1). For each set of compound pull-downs, we included a control experiment using ATP competition (4 mM) to confirm kinase capturing via the ATP binding cleft and samples were analysed by multiplexed quantitative mass spectrometric analysis. The number of identified peptides per kinase was used as a semi-quantitative indicator of the amount of protein captured. ATP-competitive binding of JAK family kinases was confirmed by immunoblotting (Supplementary Fig. 2). From this dataset, we selected nine capturing compounds (summarized in Fig. 1a) with complementary kinase selectivity to achieve maximal kinome coverage. Two of these, an analogue of PD173955^29^ (**5**) and Bisindolylmaleimide-VIII (**7**) were already included in our original kinobeads. **2** is based on CTx-0294885 with a modified linker^30^ a compound that has been recently described as a very efficient kinase capturing tool^31^. **6** is the CDK inhibitor SNS-032^32^ and **9** is the slightly modified pan-AKT inhibitor GSK690693^33^. In addition, the set includes common kinase inhibitor scaffolds: 4-Aryl-pyrimidine (**1**)^34^, Diaminopyrimidines (**2**, **3** and **4**) and Amino-pyrazine (**8**)^35^.

The direct comparison of the numbers of identified peptides per kinase from individual compound matrices (Fig. 1b) shows, that many kinases are captured by more than one compound and that the compounds display clear differences in their preference for kinase families as well as overall selectivity. Some of the diaminopyrimidines (**2**, **3**, **4**) show no preference for any kinase family. In contrast **1** and **5** preferably capture tyrosine kinases and tyrosine kinase-like proteins. **6** shows a clear preference for CMGC kinases. Interestingly, **8** enriched kinases from all kinase families but only very weakly captured tyrosine kinases. Finally, **7** and **9** both enrich only very few kinases without a clear preference for any kinase family but addition of these compounds improved the capturing of PKCs (**7**) and PKA (**9**).

### Characterization of the new kinase-capturing matrix

To characterize the new kinobeads, we determined the extent to which this bead matrix depletes kinases from cell extracts. For this experiment lysate is initially incubated either with blocked beads or with kinobeads, both non-bound fractions are again incubated with fresh kinobeads and the abundances of captured proteins from the first and the second incubation step are compared. We performed incubation in duplicate using the same human lysate mix as above. In addition, two experiments using competition with a mixture of all nine compounds at concentrations of 5 and 50 µM each were performed. These six experiments were combined into one multiplexed experiment using isobaric mass tags.

**Figure 2:**
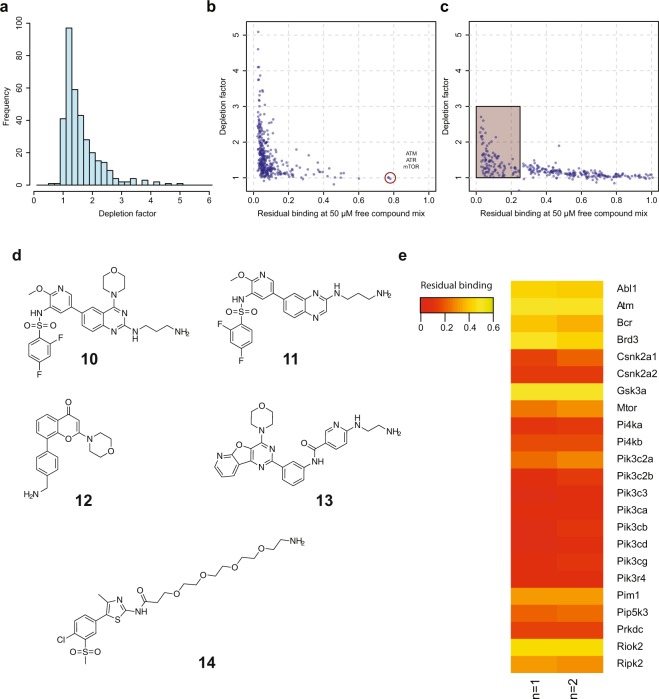
Characterization of binding, depletion and competition properties of the new kinobeads mix. (**a**) Distribution of depletion factors for all identified kinases from an experiment using a lysate mix consisting of K562, HEK293 and human placenta shows that majority of kinases are only weakly depleted. (depletion factor F = 1 + [B]/K_d_B, with [B] indicating the bead ligand concentration and K_d_B being the respective dissociation constant). (**b**) Scatterplot displaying residual binding of all identified kinases on the beads when 50 µM free compound mix was present versus the depletion factor for kinases. Target proteins should ideally be in the left corner. Kinases in the right corner (red circle), which are neither depleted nor competed, bound unspecifically to the matrix. (**c**) Same plot as in b but for all non-kinase proteins. Square indicates non-kinase proteins with less than 25% residual binding at 50 µM free compound mix. (**d**) Compounds used for the lipid kinobeads. (**e**) Heatmap displaying residual binding for all kinases captured with the lipid kinobeads (n = 2, see also Supplementary Table 4).

We could quantify 346 human kinases (out of nearly 3000 identified proteins) and determined their depletion factors in pull-down experiments (Supplementary Table 2). The depletion factor of a protein by the kinobeads matrix is calculated as the ratio between the second and the first binding experiment. For the majority of kinases we observed depletion factors below 3 (Fig. 2a,b), which indicates that only a fraction of the available kinase is removed from the equilibrium between free compound and its target. In addition, almost all kinases could be competed from the beads with a mixture of the unbound capturing compounds dissolved at 50 µM each (Fig. 2c). This demonstrates that kinases are specifically captured and retain their proper fold in the lysate during the incubation. However, the kinases ATM, ATR, MTOR and to a lesser degree DNAPK could not be displaced from the beads, which argues for either an unspecific binding mode or protein aggregation post binding to the beads. For these three kinases, it was impossible to determine apparent dissociation constants in this setup and as a consequence they were excluded for all further analyses.

Interestingly, many non-kinase proteins also bound to the beads and could be competed off the beads by the compound mix (Fig. 2c). To identify proteins that show similar binding characteristics as kinases we used a cut-off of 25% residual binding at 50 µM free compound mix. We found that more than 90% of the identified kinases had a residual binding of less than 25% and identified 393 additional proteins that fulfilled this criterion. Amongst these proteins are known kinase interactors suggesting indirect binding as part of kinase complexes, e.g. cyclins which bind to CDKs or Ras association domain-containing proteins which bind to STK kinases^36^. Moreover, several proteins for which no kinase association has been described also bound to the kinase inhibitor matrix. The kinase inhibitor off-targets Nqo2^11^ and Ferrochelatase^30,37^ have recently been described to directly bind to kinase inhibitors. The acyl-CoA dehydrogenases ACAD10 and which both contain a dehydrogenase domain and a kinase like domain^38^ are also likely direct kinase inhibitor binders, interacting with the kinase inhibitor matrix via their kinase-like domain. The dCTP pyrophophatase 1 (XTP3TPA) hydrolyzes dNTPs with a preference for non-canonical dNTPs to reduce the intracellular levels of non-canonical nucleotides and has been proposed as a target for cancer therapy^39^. Non-kinase proteins captured by the kinobeads often contain a nucleotide binding domain. These proteins are of general interest as they have the intrinsic capacity to bind to kinase inhibitor-like scaffolds and should be considered when assaying kinase inhibitor selectivities^40^.

The observation that the lipid kinases ATM, ATR and MTOR were captured but not competed on our kinobeads matrix, in conjunction with a weak coverage of the lipid kinase family motivated us to evaluate means to cover this target class in a separate bead set. Building on a previously described bead set based on PI3 kinase inhibitors that also enrich atypical and lipid kinases^41^ we investigated several PI3K scaffolds in this work, and formed an additional bead set consisting of five unspecific lipid kinase inhibitors (Fig. 2d). This set enables specific capturing and competition of 15 lipid kinases including PIP5K3 and 8 further proteins with an annotated kinase domain, including the afore-mentioned atypical kinases ATM and MTOR. This alternative lipid kinobeads capturing matrix allows profiling of potential lipid and atypical kinase inhibitors and can be added to protein kinase inhibitor profiling efforts for increased kinome coverage. All subsequent experiments have been performed solely with the protein kinobeads matrix.

**Figure 3:**
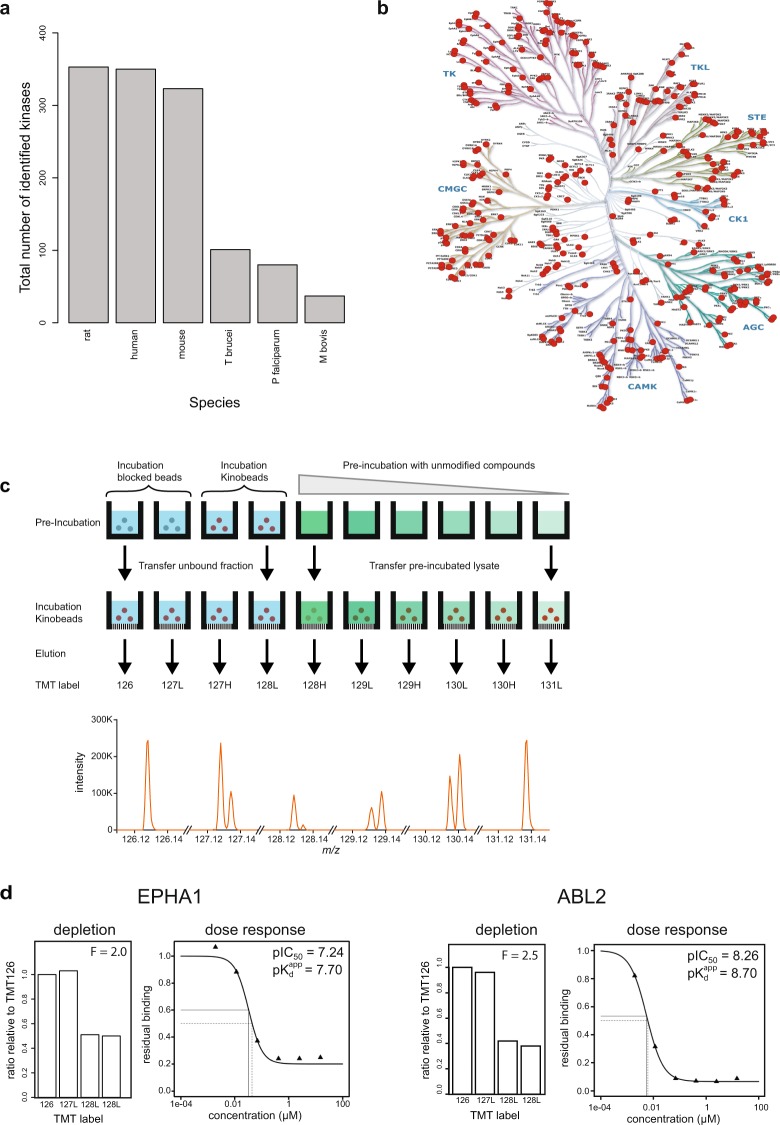
Kinome coverage and experimental setup to determine apparent dissociation constants. (**a**) Number of all kinases identified per species (see also Supplementary Table 2). (**b**) Distribution of identified human kinases on the kinome tree demonstrates good coverage of all major kinase families. (**c**) Schematic displaying the new combined experiment format to determine potency to a free compound and bead-derived depletion factor in the same experiment (**d**) Representative data for (**c**) using a lysate mixture of K562, HEK293 and human placenta and dasatinib for competition (see also Supplementary Table 5).

To further evaluate the kinome coverage of the new protein kinobeads, we performed pull-downs using lysates from various species and determined the kinome coverage. In addition to human cell lines and tissue, we tested tissue extracts of frequently used preclinical model systems (mouse, rat), lysates of disease-causing human parasites (*trypanosoma brucei* and *plasmodium falciparum*) and the intracellular pathogen *mycobacterium bovis*. We identified 359 rat protein kinases (out of 521 predicted), 352 human protein kinases (518 predicted) and 324 mouse protein kinases (540 predicted). Further, we found 101 kinases from *trypanosome* and 80 kinases from *plasmodium* and 37 kinases from *mycobacterium bovis* that bound specifically to the kinobeads matrix (Fig. 3a) underscoring the suitability of kinobeads profiling to support drug discovery in these organisms and to differentiate kinase-binding in the pathogen vs. the host species^42–44^. The slightly higher kinome coverage observed for rat (359 kinases) might be attributed to the higher number of tissues used from rodent species, which might provide greater protein diversity due to multiple cell types in one tissue compared to the selection of a few cell lines. The choice of tissue/cell type has indeed a major influence in the observable kinome as each of the different input materials identifies unique kinases (Supplementary Table 2). To visualize the kinome coverage, we marked all identified human kinases in a phylogenetic kinome tree (Fig. 3b). Almost all branches of the kinome tree are covered which underscores the broad selectivity of these beads for the kinase family. Compared to the previous version of kinobeads the most notable difference is the by more than 50% increased coverage of the AGC, the CMGC and STE subfamily (Supplementary Table 3).

For selectivity profiling of underivatized kinase inhibitors, competitive binding experiments are performed with kinobeads in which the reduction of matrix binding of targets of the tested kinase inhibitor is measured as a function of inhibitor concentration by means of quantitative mass spectrometry. Measured IC_50_ values can be influenced by the kinobeads matrix essentially following the Cheng-Prusoff relationship^45^. In order to determine by how much the binding of kinases to the kinobeads influences the binding equilibrium between free kinase inhibitor and its target kinases and to calculate apparent dissociation constants (K_d_^app^), depletion factors F (defined by 1 + [B]/KdB) are determined for each kinase using sequential binding experiments. Depletion factors are predominantly a function of the affinity of inhibitors to captured proteins and the concentration of the tagged ligands. Since kinases can be present in different activation states or different isoforms which can change their affinity towards the capturing matrix, combining the determination of the depletion factor and the dose-response into a single experiment from the very same lysate ensures that the correct depletion factors are used for IC_50_ correction^14^. We therefore employed the recently introduced 10-plex tandem mass tags (TMT10)^46^ to combine the analysis of depletion and competition into a single experiment (Fig. 3c). A major advantage here is that a depletion factor is determined for every identified kinase, whereas the separate analysis might lead to missing depletion factors due to the random sampling of the mass spectrometer. Example data for the broad specificity kinase inhibitor dasatinib are displayed in Fig. 3d.

### Miniaturization and automation of the kinobeads profiling workflow

Chemical proteomics experiments usually require substantial quantities of cell extracts corresponding to several milligrams total protein per sample^13,43^. For an experiment with 7 compound concentrations and one vehicle control we have previously reported consumption of a total of 40 mg protein^10^. Such amounts are easily accessible for one or several test compounds when immortalized cell lines are used, corresponding to approximately 10 confluent 15 cm dishes per experiment. However, they are prohibitive for larger profiling campaigns, work with scarce primary materials derived from tissues or blood cells of individual animals or even patients.

**Figure 4:**
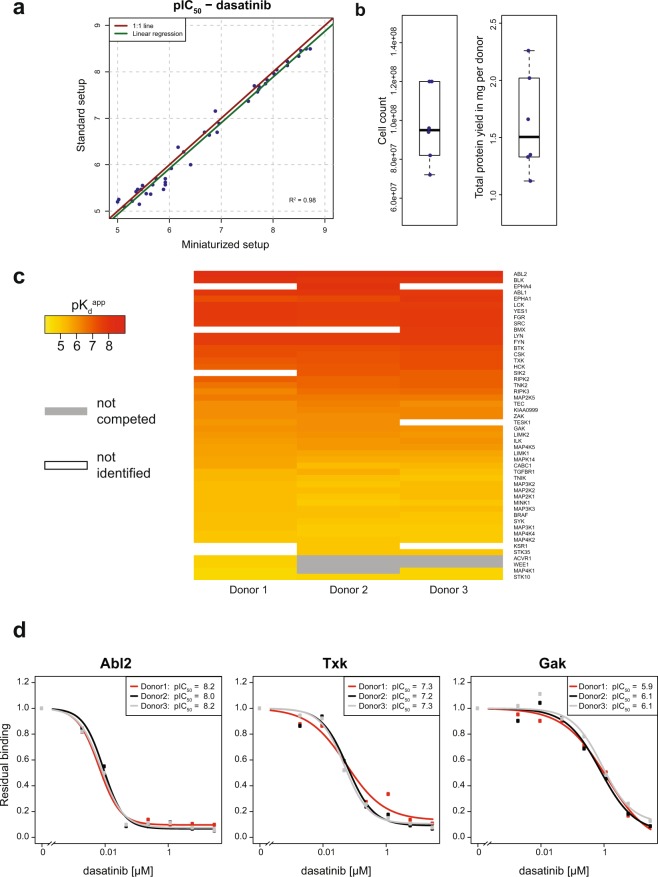
Miniaturization of the kinobeads assay enables experiments using small amounts of human primary material. (**a**) Comparison of pIC_50_ values for dasatinib determined from kinobeads pull-downs in standard setup and 1:20 miniaturized setup. Red line is 1:1-line, green line is linear regression. (**b**) PBMC cell count and total protein yield derived from 50 ml individual donor blood (n = 6) (**c**) Heatmap displaying apparent potencies for kinases from PBMC lysates generated from 50 ml blood of three individual donors for the kinase inhibitor dasatinib. (**d**) Representative dose-response curves for three dasatinib targets spanning a broad potency range (see also Supplementary Table 4).

We hypothesized that the enhanced kinobeads in combination with the high sensitivity of recent mass spectrometry instrumentation should enable developing a substantially downscaled assay while still retaining high kinome coverage. To miniaturize our chemoproteomics workflow, we reduced the protein amount used per data point to 250 µg protein corresponding to a downscaling factor of 20 compared to previous work^10^. To keep the kinase capturing efficiency and depletion characteristics comparable to previous experiments, we kept the protein concentration at 5 µg/µl and the bead-to-protein ratio constant, resulting in a reaction volume of 50 µl and 1.75 µl beads. At these low volumes, working in tubes, columns or even 96-well plates is impractical and compromises assay quality, as the surface to volume ratio in these vessels is very high, and mixing by shaking would hardly be possible. We therefore switched to 384-well filter plates with a maximum volume of 100 µl, which provides an ideal reaction container for these pull-downs. We tested this setup using the broad specificity kinase inhibitor dasatinib. Using a mixture of human cell and tissue extracts, we could identify more than 280 protein kinases with excellent reproducibility (Supplementary Table 5, Supplementary Fig. 3) in this miniaturized setup using the above described new kinobeads in dose-dependent competition binding experiments. The majority of the identified dasatinib targets yielded good quality dose-response curves and obtained pIC_50_ values obtained showed excellent correlation (R^2^ = 0.98) with a kinobeads profiling results obtained from 5 mg protein per data point (Fig. 4a).

To assess applicability to human blood samples and potentially patient material, we isolated PBMCs from 50 ml of human donor blood, a typical amount available in a clinical setting, and prepared protein extracts thereof. Each blood sample yielded approximately 100 million cells from which we could obtain a total of 1.6 mg protein (Fig. 4b). To perform 8-plex proteomics experiments, 150 µg protein were used per data point. We measured kinome selectivity profiles of dasatinib using lysates generated from three different donors. In each experiment more than 170 protein kinases were detected and affinities to dasatinib were highly reproducible between the individual donors (Fig. 4c, Supplementary Fig. 4, Supplementary Table 6). This demonstrates that chemoproteomics in general and the kinobeads platform in particular are applicable to primary human material.

To perform these experiments at higher throughput while maintaining high assay quality, we automated the workflow. Analogous to a screening setup which has been published previously^41,47^, we utilized a liquid handling workstation to perform the majority of liquid transfer steps.

### Investigation of the selectivity of clinical Jak inhibitors

**Figure 5:**
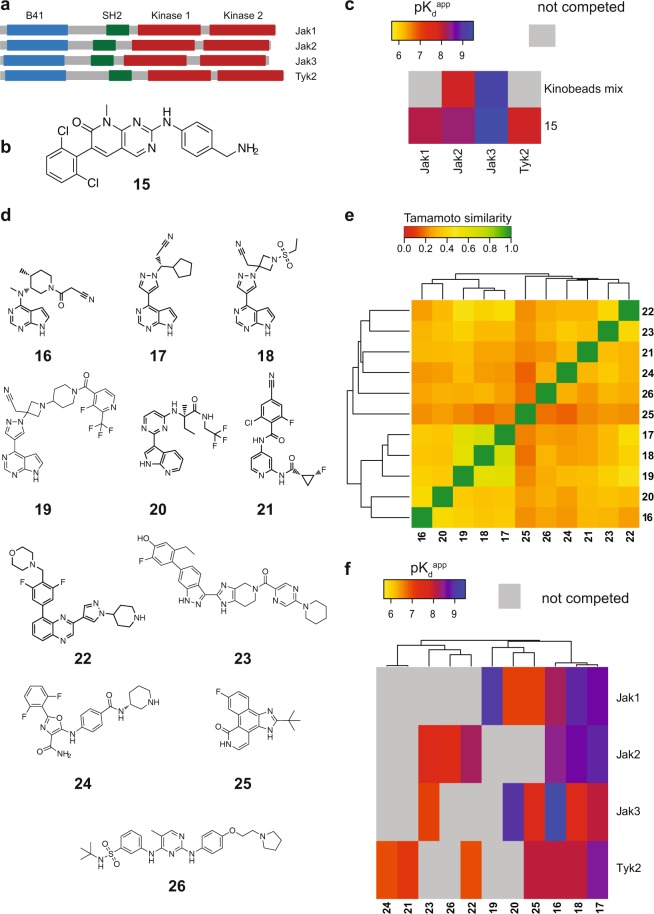
Chemoproteomic analysis of the JAK kinase family. (**a**) Overview of the domain organization of the JAK family. Two kinase domains are present in all members of this kinase family. (**b**) Chemical structure of capturing compound **15** used to enrich all JAK family members via their active kinase domain. (**c**) Determination of apparent pK_d_s using a chemoproteomic workflow with antibody readout for the marketed kinase inhibitor tofacitinib **17** (which only binds to the second (active) kinase domain). Capturing tool was either the new kinobeads mix, or **15**. (**d**) Chemical structures of all JAK family inhibitors used in this study. (**e**) Tanimoto similarities for the structures from. (**d**,**f**) Determination of apparent dissociation constants of 11 JAK kinase family inhibitors for the JAK kinase family.

The JAK kinase family, consisting of the protein kinases JAK 1–3 and TYK2, are attractive targets for pharmacological intervention to treat inflammatory and autoimmune diseases, especially rheumatoid arthritis, as well as several myeloproliferative disorders^19–22^. A peculiarity of JAK family kinases is the presence of two kinase domains, an active and a pseudo kinase domain^48^ (Fig. 5a). As the kinobeads workflow utilizes the principle of binding and competition, we tested all four JAK kinases for a unimodal binding mode via their active kinase domain to the kinobeads matrix. Competition experiments using the pan-JAK inhibitor tofacitinib (compound **15)**, which has been described to bind to active kinase domains of JAK kinases but not to the pseudokinase domain of JAK1^49^ revealed inhibition of binding for JAK2 and JAK3 but not for JAK1 and TYK2 (Fig. 5c). Assuming that this finding is mediated by one or more kinobeads compounds to the pseudokinase domain of JAK1 and TYK2, individual compounds were tested in a competition binding experiment again with tofacitinib as test compound (Supplementary Fig. 2). Compound 4 and to a lower extent compound 8 were identified to bind to the pseudokinase domain of both JAK1 and TYK2. However, when using an affinity matrix created with compound **15** (Fig. 5b), all members of the JAK kinase family could be competed (Fig. 5c, Supplementary Fig. 2) at nanomolar affinities comparable to published data^27,49^, JAK2 (pK_d_^app^ = 8.5) JAK3 (pK_d_^app^ = 9.5) JAK1 (pK_d_^app^ = 8.3) and TYK2 (pK_d_^app^ = 8.1).

We selected a set of 11 JAK kinase inhibitors, spanning marketed, clinical, and tool compounds (Fig. 5d) to generate a better understanding about selectivity within the kinome space and within the Jak family in particular. Commonly used names for the selected compounds are: **16** tofacitinib, **17** ruxolitinib, **18** baricitinib, **19** INCB-39110, **20** decernotinib, **21** Genetech Tyk2, **22** NVP-BSK805, **23** Pfizer indazole, **24** Sareum Tyk2, **25** Pyridone 6, **26** fedratinib. The Tanimoto similarity suggested high chemical diversity within this compound set (Fig. 5e). A cluster consisting of the pyrrolo-pyrimidines tofacitinib, ruxolitinib, baricitinib, decernotinib, and INCB-39110 showed some degree of similarity, which is mainly driven by the core of the molecules with major differences in their auxiliary groups. All other compounds displayed low similarity between each other, with Pyridone 6 being unlike any other compound in the set. In addition to our mass spectrometry-based kinobeads assay, we used sepharose-coupled compound **15** and immunodetection to generate affinity data for the active kinase domains of the complete JAK family kinases.

We applied this workflow (mixed inhibitor matrix with proteomics readout for kinome and matrix with compound 15 with dotblot assay for JAK family kinases) to profile this set of JAK kinase family inhibitors in a donor-pooled human PBMC lysate. PBMCs consist of a mix of circulating immune cells covering many of the target cells of JAK kinase inhibitors. Affinities within the JAK kinase family were largely in line with reported potencies of the selected compounds (Fig. 5f, and Suppl. Table 7). As expected, the pan-JAK inhibitors tofacitinib, ruxolitinib and baricitinib bind to all members of the JAK kinase family with comparable affinities. But for each of the JAK family kinases, at least one inter-family selective inhibitor can be found in this inhibitor collection. INCB-39110 is very potent for JAK1 (pK_d_^app^ of 8.9) and none of the other JAK kinase members was competed. NVP-BSK805 shows a window of 1.3 log units for JAK2 to the next JAK kinase member TYK2. Decernotinib has high selectivity window between JAK3 and JAK1 with nearly two orders of magnitude difference in binding strength. TYK2 was the only JAK kinase member that was competed by both TYK2 tool compounds, although at relatively high compound concentrations.

**Figure 6:**
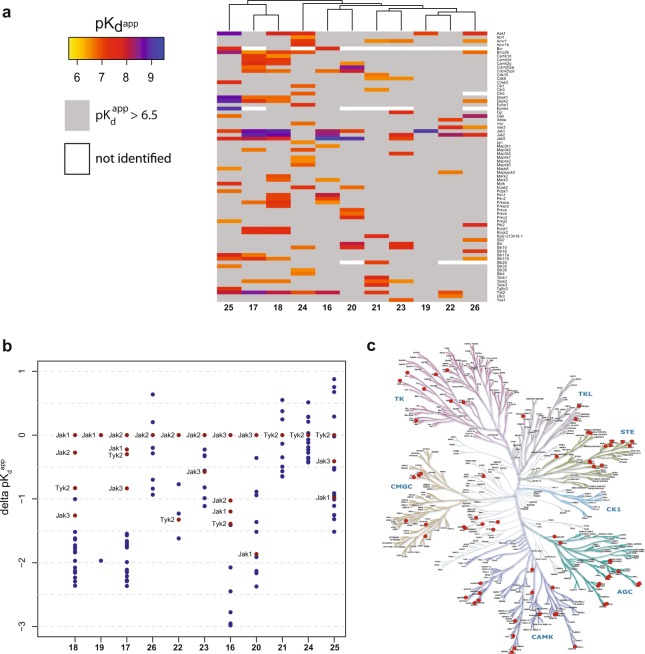
Kinome-wide selectivity of JAK kinase inhibitors determined by kinobeads experiments using PBMC lysate. (**a**) Heatmap displaying all competed kinases from kinobeads experiments. Experiments were done in duplicate using two different concentration ranges; displayed value is mean of calculated apparent pK_d_s. Only kinases are displayed for which at least one inhibitor showed an apparent pK_d_ above 6.5 (**b**) Potency windows (delta apparent pK_d_) between the most potently hit JAK kinase (set as reference) to all other competed kinases for all investigated JAK kinase inhibitors. JAK kinases are marked in red, all other kinases are marked in blue. (**c**) Distribution of all identified JAK kinase inhibitor off-targets over the kinome tree. (see also Supplementary Table 7).

Kinobeads-based profiling experiments with mass spectrometry detection performed in PBMC lysates assessed the selectivity against 250 protein kinases. Figure 6a (and Suppl. Table 7) summarizes all kinases for which at least one inhibitor had a pK_d_^app^ of greater than 6.5. This cutoff was chosen because lower potency targets are mainly not relevant for these highly potent JAK inhibitors. We investigated for each inhibitor the potency window between the most potently bound JAK kinase family members to all other bound kinases (Fig. 6b). For the three marketed kinase inhibitors – baricitinib, ruxolitinib and tofacitinib – JAK kinases were at least 10-fold more potently bound than the most potently bound non-JAK kinase. The pan JAK inhibitors ruxolitinib and baricitinib, sharing the same pyrazole-pyrolopyrimidine scaffold, have many off-targets in common. Interestingly, the overlapping kinase off-targets of these two compounds are not tyrosine kinases but belong to the AGC kinase family (ROCK1, ROCK2, PRKACA, CDC42BPA, CDC42BPB), CAMK kinase family (CAMK1D, CAMK2D, CAMK2G, DAPK1, DAPK2) and CMGC kinase family (BMP2K). INCB-39110 is a highly selective JAK1 inhibitor, with a window of more than two orders of magnitude to the first identified off-target. For the JAK inhibitor fedratinib, which recently failed in phase III clinical trials^50^, a large number of additional kinases were found to be bound with similar or even higher potency as the primary target. The frequently used tool compound Pyridone 6 (Calbiochem JAK1) hits a large number of off-targets, mainly CAMK kinases, like DAPK1, 2 and 3 but also CHEK2 or MYLK and has no window at all between the assumed main target and off-targets. The two TYK2 tool compounds (**21** and **24**) demonstrated that selectivity for TYK2 compared to the other JAK kinase members is possible. However, these two molecules are still at early phases in discovery and display low potency and a large number of kinase off-targets. Interestingly, AGC kinases were among the frequently engaged targets of the investigated JAK inhibitors. For oncology indications, activities against MRCK and ROCK kinases are of substantial interest, as these kinases have been shown to play a crucial role in cell invasion^51^ and metastasis^52^. The highest affinity towards MRCK kinases was observed for decernotinib with pK_d_^app^ of 8.0 and 8.6 for MRCK α and β, respectively.

To generate a better view of the kinome wide distribution of all identified off-targets which are bound by any member of this set of JAK family inhibitors, we visualized all off-targets on the kinome tree (Fig. 6c)^3^. Interestingly, there are only very few kinase off-targets in the tyrosine kinase space, and off-targets outside this sub-group are distributed over the complete kinome tree. This finding indicates that although inhibitors have been optimized for specificity within the tyrosine kinase space, members of other kinase classes are often bound. The Numb-associated kinases (NAKs) AAK1 and BMP2K (Bike) are the most frequently encountered off targets and were bound by almost half of the tested compounds. The binding of some JAK inhibitors including Ruxolitinib, Baricitinib and Fedratinib to these kinases had been desmonstrated previously^53^ and could be explained by conserved binding modes between NAKs/JAKs. The data presented here provides the first comprehensive view of JAK kinase inhibitor selectivity in PBMCs and demonstrates the utility of an unbiased kinome-wide profiling to comprehensively characterize kinase inhibitor selectivity.

## Discussion

Chemoproteomics-based profiling enables measuring target selectivity and engagement of endogenous protein kinases from primary, patient-derived material. However, the application of the approach to larger sample cohorts and primary cell systems has thus far been limited by the large sample demand. We made use of a diverse collection of unspecific kinase inhibitors to generate an improved kinobeads capturing matrix. In combination with the latest mass spectrometry instrumentation and optimized experimental procedures, we improved the kinome coverage compared to previous reports while using only 5% of the protein amount per data point. In combination with automation of liquid handling, a robust medium-throughput protocol was developed. The throughput and quality which is now accessible enables profiling of larger compound sets. For example, all molecules synthesized during lead optimization phase could be screened for their promiscuity to guide chemistry not only by affinity to the primary target, but also by kinome-wide selectivity. A recent report utilized systematic profiling of clinical kinase inhibitors^14^ and exemplified the power of large scale compound profiling to identify rationales for drug repurposing.

Another unique feature of this chemoproteomics-based profiling approach is that it is easily extended beyond human kinases. A similar kinase coverage as for humans could be obtained for mouse and rat. In general, only a low number of kinase assays for species are offered as part of commercial kinase panels. In particular, this also holds true for non-mammalian species where the new bead set offers unprecedented coverage of parasite kinomes like trypanosoma, plasmodium falciparum and leishmania, which should be very valuable for the discovery of kinase inhibitors specifically targeting parasite kinases^42,43^.

We applied the improved kinobeads and the miniaturized chemoproteomics enrichment protocol to characterize a representative collection of JAK kinase inhibitors. Compound selectivity data within the JAK kinase family and kinome-wide will help select the appropriate tool molecules for biological interrogation of the JAK pathways. Chemical probes are a powerful tool for modulation and interrogation of a pathway, however it is crucial to ensure that the probe is sufficiently well characterized and inhibits only the primary, intended target in the applied concentration range. Misinformed usage can lead to severe misinterpretation of observed phenotypes^54^. Here, we provide not only selectivity information for a collection of JAK kinase inhibitors, but also their affinities to targets and off-targets. This will support the decision on which inhibitors to use and also influence the tested concentration ranges. Based on this data, we would recommend INCB-39110 as JAK1 selective inhibitor and NVP-BSK805 as JAK2 selective inhibitor. Decernotinib shows very high selectivity for JAK3, however, its off-target activities demand careful data interpretation. Ruxolitinib proved to be the preferable pan JAK inhibitor in our hands. The compounds which we selected as TYK2 selective do indeed show selectivity within the JAK family, however, due to their broad off-target space we cannot recommend their application for interrogation of biological processes. Finally, we strongly discourage usage of fedratinib or pyridone 6 due to the large number of identified kinase off-targets. As expected, kinase off-targets could be identified for all tested compounds. Interestingly, the majority of identified kinase off- targets were not protein tyrosine kinases but were distributed over the complete kinome tree. This finding demonstrates that the high conservation of the kinase domain with only small differences in the binding pocket increases the likelihood that kinase inhibitor off-targets within the kinase family can be phylogenetically distant to the primary target. As a consequence, unbiased profiling of a major fraction of the kinome is necessary to accurately characterize kinase inhibitor specificity. This data can be utilized for predicting drug safety and potentially also enable drug repurposing. In addition, the identification of off-targets of pharmacological interest provides starting points for chemistry to generate new probes or even drugs. For example, PKN1 has been recently identified as an off-target of tofacitinib and has then been used as a starting point for chemical optimization to generate PKN1 inhibitors^55^. We could confirm this interaction and in addition we identified decernotinib as a highly potent inhibitor of MRCK alpha and beta. The collection of JAK inhibitors presented here also adds some initial SAR which can be utilized for further optimization.

In summary, we describe an unbiased quantitative kinase inhibitor profiling workflow in a setting of limited primary human material. Previous work has demonstrated that chemoproteomics from limited sample amounts is possible, but mainly focused on qualitative analysis. Our data however quantitatively assessed compound selectivity by acquiring dose-dependent binding data across the kinome for each individual donor. Such an assay could enable the prediction of treatment response and fine tuning of drug doses based on an individualized functional read-out by providing direct evidence for target engagement.

## Material and Methods

### Compounds

All compounds used in this study were either synthesized in house or purchased from commercial vendors (see Supplementary Material for chemical synthesis).

### Cells and lysis

HEK293 and K562 cells were derived from DSMZ and ATCC, respectively and cultured according to vendors instructions. Human placenta was supplied by Biopredic international (Saint Grégoire, France). Human peripheral blood monocytic cells (PBMCs) were isolated using Leucosep tubes (Greiner Bio-One) according to manufacturer’s instruction either from buffy coats supplied by the German Red Cross (Deutsches Rotes Kreuz, Mannheim) or from 50 ml blood aliquots donated by healthy volunteers. The dog cell line DH82 was purchased from ATCC and cultured according to manufacturer’s instructions. Mouse tissue was obtained from Charles River (Sulzfeld, Germany), rat tissue was obtained either from Charles River (Sulzfeld, Germany), Heidelberg Pharma (Heidelberg, Germany).

Human biological samples were sourced ethically, all donors provided informed consent, and research use was in accordance with the terms of the informed consents under an IRB/EC approved protocol (REC approval no. 2007-036-f#A5). Ethical approval was granted by the “Ethik-Kommission bei der Landesärztekammer Baden-Württemberg”, Germany.

Large scale preparation of cell extracts from frozen cell pellets was done as described previously^43^. Briefly, frozen cell pellets were homogenized in 2 pellet volumes lysis buffer (50 mM Tris-HCl, 0.8% NP-40 ((octylphenoxy poly(ethyleneoxy)ethanol)), 5% glycerol, 150 mM NaCl, 1.5 mM MgCl_2_, 25 mM NaF, 1 mM sodium vanadate, 1 mM DTT, pH 7.5). One complete EDTA-free protease inhibitor tablet (Roche) per 25 mL was added. The sample was dispersed using a Dounce homogenizer, kept rotating for 30 min at 4 °C, and centrifuged for 10 min at 20 000 *g* at 4 °C. The supernatant was centrifuged again for 1 h at 145 000 *g*. The protein concentration was determined by Bradford assay (BioRad), aliquots were snap frozen in liquid nitrogen and stored at −80 °C.

Frozen cell pellets (small scale) were homogenized in 2 pellet volumes lysis buffer supplemented with protease inhibitors (see above) by pipetting the cell suspension 20x up and down using a 200 µl pipette and gel loader tips (Steinbrenner, #952-200 G). Crude lysate was centrifuged for 1 h at 100 000 g at 4 °C. The protein concentration was determined by Bradford assay (BioRad), and lysates were snap frozen in liquid nitrogen and stored at −80 °C.

### Kinase enrichment

Amino-functionalized compounds were coupled to NHS activated Sepharose (GE Healthcare) at a coupling density of 1 mM in DMSO as described before^11^. Pulldowns in the largescale format were performed as described previously^11^, with minor modifications. Briefly, in a 96 deep-well plate (Thermo Scientific, #AB-0932) one millilitre of the specified lysate (mix) (5 mg/mL protein concentration) was incubated with the respective inhibitor for 45 min at 4 °C over a range of concentrations followed by incubation with kinobeads in a 96 deep well filter plate (Porvair, #240002) for 1 h on an end-over-end rotator at 4 °C. Beads were thoroughly washed in two steps with lysis buffer containing 0.4% and 0.2% IGEPAL CA-630 (octylphenoxy poly(ethyleneoxy)ethanol), respectively, and eluted with 50 μL of a 2 × LDS sample buffer supplemented with 50 mM DTT.

Miniaturized pulldowns were performed in 384 well (Greiner Bio-one, #781280) and 384 well filter plates (Merck Millipore, #MZHVNOW50) and beads as well as protein amount were downscaled by a factor of 20.

Automated miniaturized pulldowns were performed analogous to miniaturized pull-downs but manual pipetting steps were transferred to a robotics platform or replaced by other appropriate instrumentation: a Bravo automated workstation (Agilent Technologies) was used for compound dilution, for the distribution of diluted compounds as well as lysate to the 384 well plate and for the transfer of pre-incubated compound-lysate mix to the 384 well filter plate. A Multidrop combi liquid dispenser (Thermo Scientific) was used for the distribution of beads to the filter plate, for bead wash and addition of elution buffer.

### MS sample preparation and LC-MS/MS analysis

As reported previously^30^, eluates were alkylated with 200 mg/mL iodoacetamide for 30 min in the dark, separated on 4–12% NuPAGE (Invitrogen) for approximately 2 cm, and stained with colloidal Coomassie. Gel lanes were cut into three slices covering the entire separation range and subjected to in-gel digestion. Peptide samples were labeled with 10-plex TMT (TMT10, Thermo Fisher Scientific, Waltham, MA) reagents^10^ in 40 mM triethylammoniumbicarbonate (TEAB), pH 8.5. After quenching the reaction with hydroxylamine, labelled extracts were combined. To increase analytical depth, some samples were either fractionated into 9 fractions using reversed-phase chromatography at pH 12 (1 mm Xbridge column, Waters)^56^ or fractionated into 5 fractions using stage-tip based SCX fractionation^57^.

Samples were dried *in vacuo* and resuspended in 0.1% formic acid in water. Aliquots of the sample were injected into an Ultimate3000 RSLCnano system (Dionex, Sunnyvale, CA) coupled to a QExactive mass spectrometer (Thermo Fisher Scientific). Peptides were separated on custom 50 cm × 100 μM (ID) reversed-phase columns (Reprosil) at 40 °C. Gradient elution was performed from 2% acetonitrile to 40% acetonitrile in 0.1% formic acid 2% DMSO over 4 h for unfractionated samples and 2 h per run for fractionated samples. Samples were online injected into Q-Exactive mass spectrometers operating with a data-dependent top 10 method. MS spectra were acquired by using 70,000 resolution and an ion target of 3E6. First mass was set at 375 m/z. Higher energy collisional dissociation (HCD) scans were performed with 35% NCE at 35,000 resolution (at *m*/*z* 200), isolation width was set to 1Thompson and the ion target for MS2 was set to 2E5.

### Peptide and protein Identification and quantification

Mascot 2.2 (Matrix Science) was used for protein identification using a 10 ppm mass tolerance for peptide precursors and 20 mDa (HCD) mass tolerance for fragment ions^41^. A 2.5 mDa mass tolerance was used for reporter ion extraction in HCD scans by an in-house-developed software. Carbamidomethylation of cysteine residues and TMT modification of lysine residues were set as fixed modifications and methionine oxidation, *N*-terminal acetylation of proteins and TMT modification of peptide N-termini were set as variable modifications. The search database consisted of a customized version of the IPI protein sequence database combined with a decoy version of this database created using a script supplied by Matrix Science.

Reporter ion intensities were read from raw data and multiplied with ion accumulation times (the unit is milliseconds) so as to yield a measure proportional to the number of ions; this measure is referred to as ion area^58^. Spectra matching to peptides were filtered according to the following criteria: mascot ion score >15, signal-to-background of the precursor ion >4, and signal-to-interference >0.5. Fold-changes were corrected for isotope purity as described and adjusted for interference caused by co-eluting nearly isobaric peaks as estimated by the signal-to-interference measure^59^. Protein quantification was derived from individual spectra matching to distinct peptides by using a sum-based bootstrap algorithm.

For dose–response inhibitor data dose–response curves were fitted using R (http://www.r-project.org/) and the drc package (http://www.bioassay.dk), as described^11^. To compensate for bead-matrix induced depletion^45^, IC50 values were corrected by an experimentally-derived depletion factor as described before^60^.

Complete results of all proteomics experiments can be found in the Supplementary Tables.

### Determination of JAK affinities using antibodies

Similar to procedures published previously^41^, potencies for JAK kinase were determined using the same pulldown procedure as described above, with minor modifications: 0.9 µl beads (compound 10 at 1 mM coupling density) were incubated with 125 µl PBMC lysate. Pull-down eluates were spotted on nitrocellulose membranes (400 nl per spot) using an automated pin-tool liquid transfer (Biomek FX, Beckman). After drying, the membranes were rehydrated in 20% (v/v) ethanol and processed for detection with specific antibodies (Jak1 (Millipore, #05-1154), Jak2 (Cell Signalling #3230),Jak3 (Cell Signaling, #3775), Tyk2 (Abcam, #ab57678)), followed by incubation with an IRDye 800–labelled secondary antibody for visualization (anti-rabbit 926-32211, anti-mouse 926-32210, LICOR (1:3,000)). Spot intensities were quantified using a LiCOR Odyssey scanner, and percentage inhibition was calculated using positive and negative controls as 100% and 0% inhibition, respectively. For western blot analysis, eluates were loaded on NuPage Bis-Tris 4–12% polyacrylamide gels and a MES SDS running buffer (Life Technologies) was used to separate the proteins in the samples. Proteins were then transferred to PVDF membranes and detected as described above.

## Supplementary information

Supplementary Information

Supplementary table 1

Supplementary table 2

Supplementary table 3

Supplementary table 4

Supplementary table 5

Supplementary table 6

Supplementary table 7

## Data Availability

All data generated or analyzed during this study are included in this article.
