# Supplementary material for: Chemical proteomics reveals target selectivity of clinical Jak inhibitors in human primary cells
Source: Sci Rep. 2019 Oct 2;9:14159. doi: 10.1038/s41598-019-50335-5 (PMC6775116; doi:10.1038/s41598-019-50335-5)
Supplement: Supplementary file 1 — Supplementary Information [file 41598_2019_50335_MOESM1_ESM.pdf]

Supplementary material for

Chemical proteomics reveals target selectivity of clinical Jak inhibitors in human primary cells

H. Christian Eberl[1*], Thilo Werner[1], Friedrich B. Reinhard[1], Stephanie Lehmann[1], Douglas Thomson[1], Peiling Chen[2], Cunyu Zhang[2], Christina Rau[1], Marcel Muelbaier[1], Gerard Drewes[1], David Drewry[3,4] and Marcus Bantscheff[1,*]

Content:

Supplementary figures 1-5

Supplementary table legends and descriptions

General chemistry methods

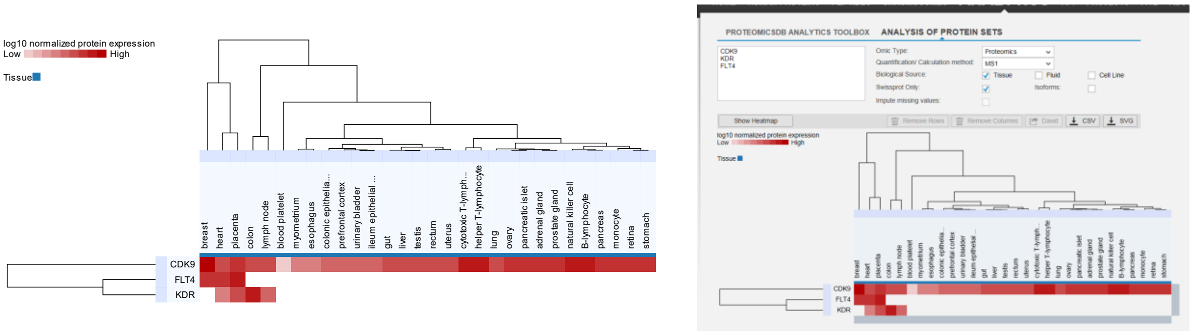

Supplementary figure 1: Expression of receptor tyrosine kinase FLT4 and KDR in human tissues. Human tissue expression reveals a very distinct expression pattern for the two kinases with human placenta providing access to both. CDK9 is included as example for a ubiquitously expressed kinase. Data taken from ProteomicsDB and visualized with tools available at the site www.proteomicsdb.org (ref 28)
https://www.proteomicsdb.org/#analytics/expressionHeatmap?proteins=CDK9%3BKDR%3BFLT4%3B&quantification=MS1&customQuantification=&biologicalSource=tissue&calculationMethod=top3&customCalculationMethod=&swissprotOnly=1&noIsoforms=1&omics=Proteomics&source=db&uuid=&datasetIds=&impute=0

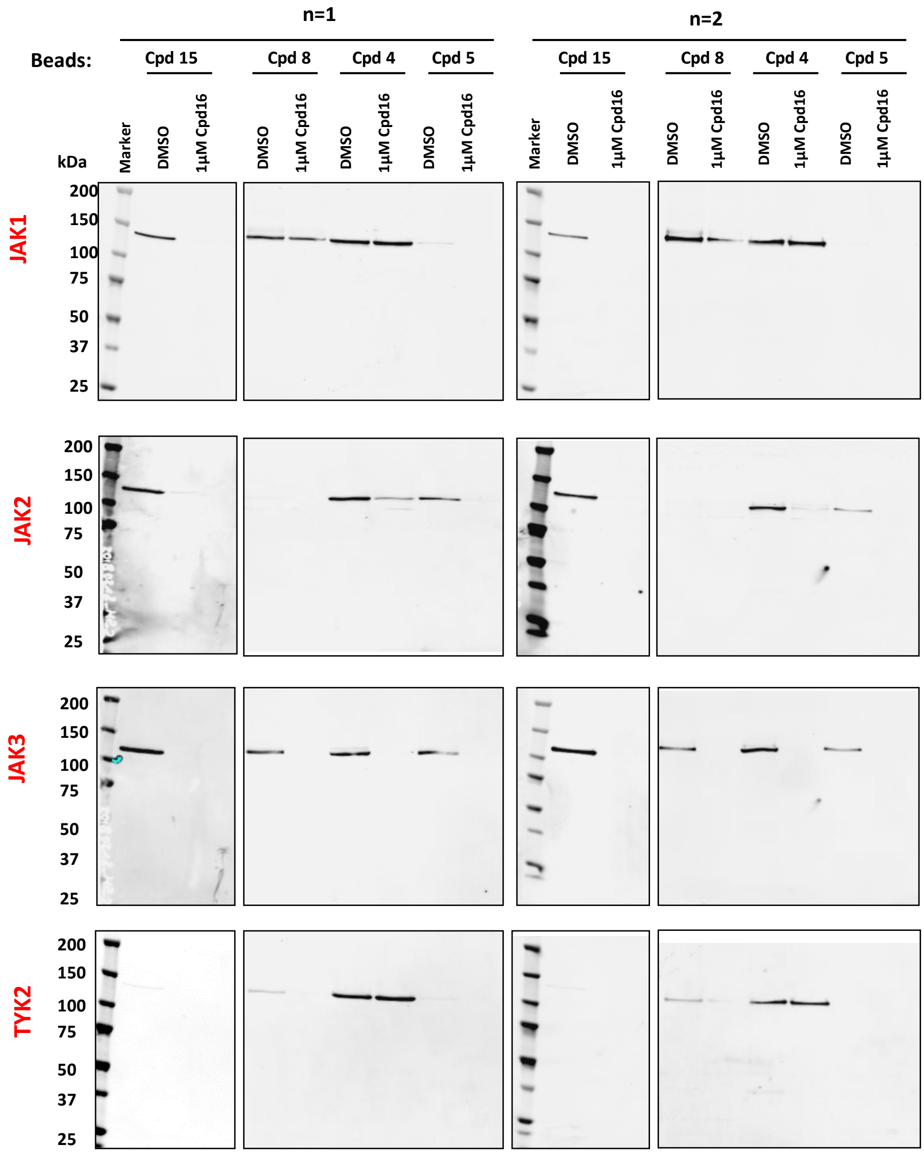

Supplementary figure 2: Binding of family of JAK kinases to immobilised compounds cpd4, cpd5, cpd8 and cpd15. The binding of JAK1 and TYK2 to Cpd4 cannot be competed by kinase domain-binder Cpd16 (tofacitinib)

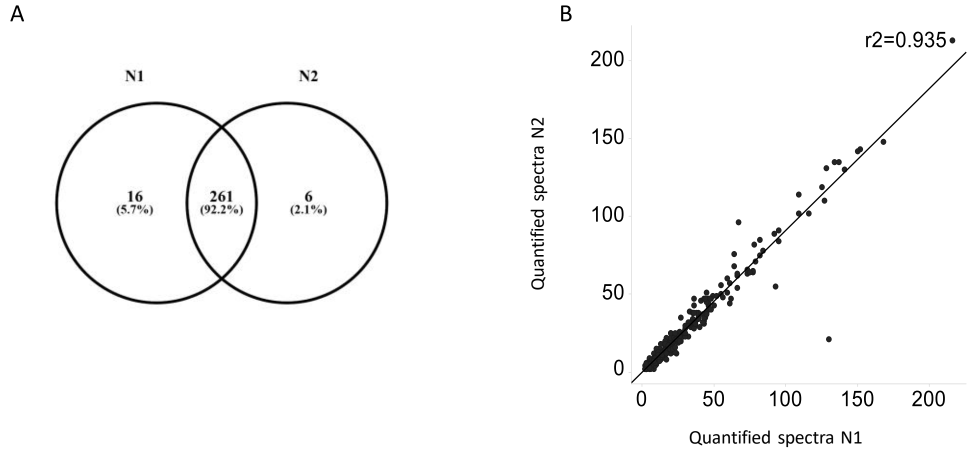

Supplementary figure 3: Reproducibility of quantification of kinases in human cell line/placenta mix (based on supplementary table 5). A) Venn diagram of two replicate experiments reveals 92.2% overlap of kinases quantified in each experiment. B) Quantified spectra of two replicate experiments show excellent reproducibility (r^2 = 0.935)

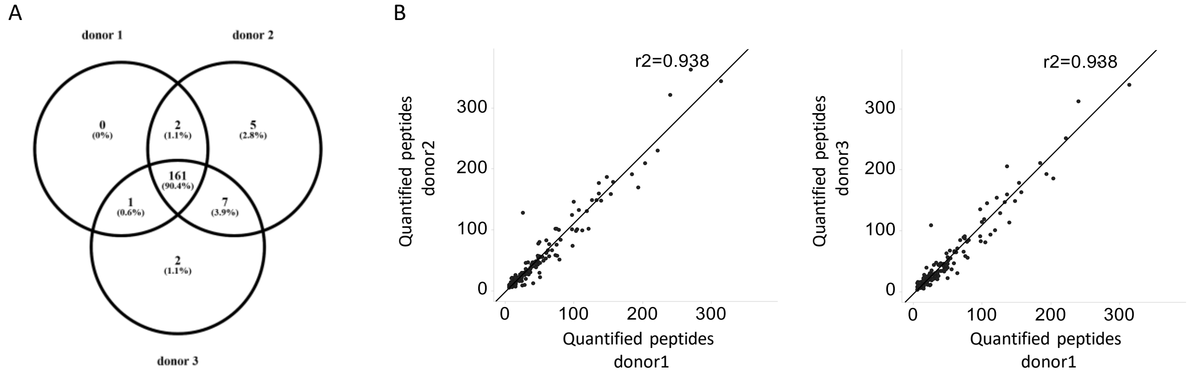

Supplementary figure 4: Reproducibility of quantification of kinases in PBMCs derived from three donors (based on supplementary table 6). A) Venn diagram of experiments based on lysates of three donors reveals 90.4% overlap of kinases quantified across all three experiments. B) Quantified peptides of donor 2 vs. donor 1 experiment (left graph) and donor 3 vs. donor 1 experiment (right graph) show excellent reproducibility (r^2 = 0.938 for both comparisons)

<u>Supplementary table legends and descriptions:</u>

Supplementary table 1: **Kinome coverage for individual capturing compounds**. This tables lists the identified kinases (kinase name, kinase family and IPI identifier) for each kinobeads capturing compound. Reported are number of identified peptides as a proxy for capturing efficiency. Each compound was profiled in a separate experiment.

Supplementary table 2: **Kinome coverage from different species**. This table list in individual tabs (each tab one species) all identified kinases (kinase name, IPI identifier, protein name) for 7 different species (human, dog, mouse, rat, P.falsiparum, T. brucei, M. bovis). All experiments are performed as depletion experiment (as described in the main text) and the table reports for each experiment the number of identified peptides, the calculated depletion factor and the competition at 50 µM free kinobeads mix. For species where multiple lysates (lysate mixes) were used, the lysate sources are indicated in the column names.

Supplementary table 3: **Comparison of kinase class coverage between old kinobeads and new kinobeads**. This first tab summarizes the kinase class coverage from comparable depletion experiments using the old kinobeads matrix (Bantscheff et all., 2007) and the new kinobeads matrix described in this manuscript. The tab ‘old kinobeads’ lists all identified kinases (IPI identifier, Gene name, Targetclass, Subclass) using the old matrix from one representative experiment, including proteinscore, total peptide to spectra matches (totalpsm), quantified spectra matches (qusm) and quantified peptide matches (qpm). The 2 columns “Binding” contain the relative abundance for each kinase in the first binding step (relative to of the two binding events), the two columns rebinding contain the relative abundance (to the first binding event) of each kinase in the rebinding step. The column “IC502KD” contains the depletion factor F calculated from these binding events. The tab “new kionbeads” contains the same information as described above, but for the new capturing matrix.

Supplementary table 4: **Kinome coverage using lipidkinobeads**. This table summarizes the kinome coverage from duplicate depletion experiments using the lipidkinobeads. For each kinase (Gene name, Targetclass, IPI identifier) the table lists for both replicates quantified peptide matches (qpm), quantified spectra matches (qusm) and proteinscore. In addition, relative abundance for binding (in n2), rebinding (in n2) as well as competition using 50 µm and 5 µM free lipidkinobeads mix and the depletion factor F (IC502KD) are reported.

Supplementary table 5: **Determination of apparent dissociation constants for dasatinib in one single experiment (n=2 data)**. This table lists all kinases (Gene name, IPI identifier and target class) identified in two replicate depletion-dose-response experiments using dasatinib. For each replicate, the table contains number of quantified spectra, depletion factor F (IC502KD), pIC_{50} and apparent dissociation constant pKd(app). ‘not competed’ indicates that no competition was observed at the highest dasatinib concentration.

Supplementary table 6: **Miniaturized kinobeads assay**. This table summarizes the results from the miniaturization of the kinobeads assay. The first tab “mini vs std_Dasatinib” summarizes the direct comparison of competition experiment using dasatinib in the standard setup versus the miniaturized setup. Kinases are listed by Gene name and number of quantified peptides and derived pIC_{50}s are listed. ‘not competed’ indicates that no competition was observed at the highest dasatinib concentration. The second tab “Dasatininb_PBMC_lysates” summarizes the results from three replicate experiments utilizing miniaturized kinobeads and PBMC lysates from three individual donors. Kinases are listed by Gene name and number of quantified peptides and derived apparent dissociation constants as well as utilized depletion factors (derived from additional PBMC experiments).

Supplementary table 7: **Kinase selectivity of 11 JAK kinase family inhibitors**. This table summarizes the apparent dissociation constants for 249 kinases derived from kinobeads experiments using pooled PBMC lysate. Apparent dissociation constants are only reported if higher than 6.5.

General Chemistry Procedures

Unless otherwise stated, all solvents and were used as purchased without further purification. Preparative mass directed high performance liquid chromatography (prep HPLC) was preformed on a XBridge BEH C18 OBD 5µm Prep Column (19 x 150 mm) at a flow rate of 30 mL/min, eluting with acetonitrile in water (0.2% formic acid as modifier). Purifications were conducted on a waters autopurification system [detectors: 3100 Mass Detector and a 2996 Photodiode Array Detector]. The purity of all final compounds was 95% or higher as determined by either, Method A: analytical high performance liquid chromatography (HPLC) using a Waters XBridge BEH C18 5µm Column (4.6 x 150 mm) at a flow rate of 1.75 mL/min with a linear gradient over 9 min (1 to 99% acetonitrile in water, 0.2% formic acid as modifier). The instrument used for analysis was a waters autopurification system [detectors: 3100 Mass Detector and a 2996 Photodiode Array Detector] or Method B: analytical ultra performance liquid chromatography (UPLC), using a Waters Acquity BEH C18 1.7 µm Column (2.1 x 50 mm) at a flow rate of 1 mL/min with a linear gradient over 2 min (3 to 99% acetonitrile in water, 0.1% formic acid as modifier). The instrument used for analysis was a waters Acquity system [detectors: Acquity SQD, Acquity ELSD, Acquity PDA]. Proton (1H) NMR spectra were recorded on a Bruker Avance 400 spectrometer (400 MHz) using DMSO-d6 or CDCl3 as solvents. Chemical shifts are given in parts per million (ppm) (δ relative to residual solvent peak for 1H).

8: 3-Amino-*N*-(4-(5-amino-6-(1-ethyl-1*H*-imidazo[4,5-*c*]pyridin-2-yl)pyrazin-2-yl)phenyl)propanamide

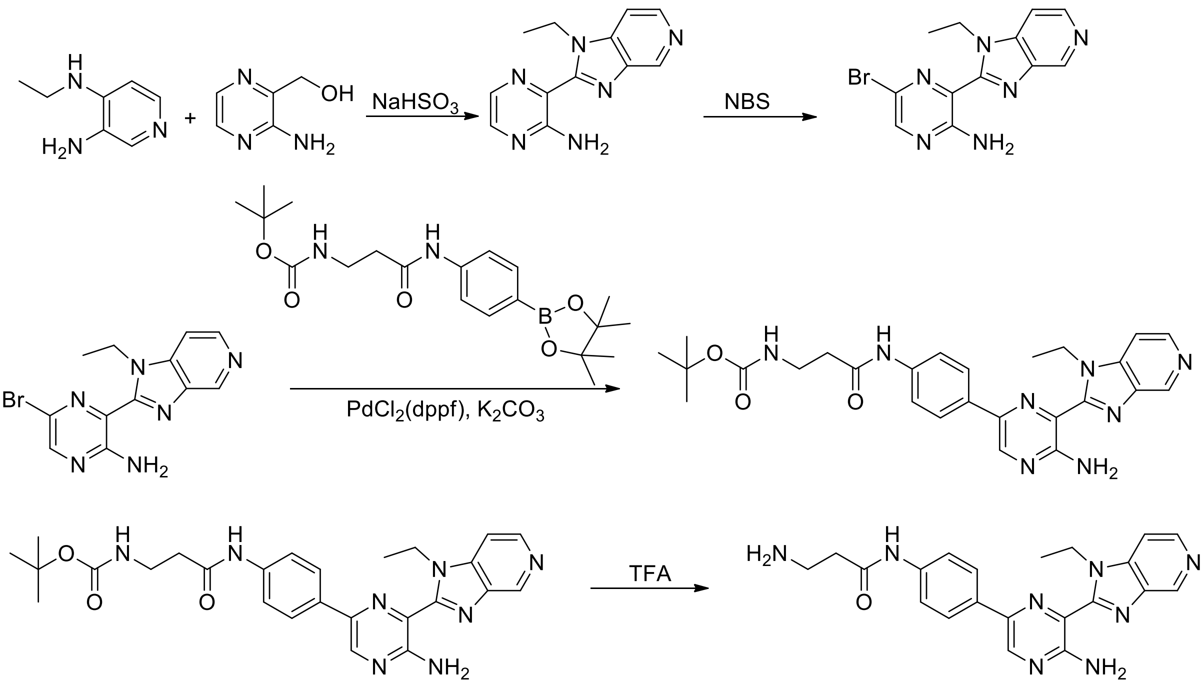

A mixture of (3-aminopyrazin-2-yl)methanol (0.90 g, 7.19 mmol), *N*-4-ethylpyridine-3,4-diamine (1.28 mg, 9.35 mmol) and sodium bisulfite (1.77 g, 17.26 mmol) in *N*,*N*-dimethylacetamide (10 mL) was heated to 200ºC for 10 min in a microwave. The mixture was partitioned between water (200 ml) and ethyl acetate (200 ml) and the aqueous phase was subsequently extracted with further ethyl acetate (2 x 200 ml).

The combined organics were washed with water (400 ml), brine (400 ml), dried over Na_2SO_4, filtered and concentrated. The residue was triturated with methanol and the solid collected by filtration. This was then further purified by column chromatography (methanol in ethyl acetate containing 1% triethyl amine). This afforded 3-(1-ethyl-1*H*-imidazo[4,5-*c*]pyridin-2-yl)pyrazin-2-amine as a mixture which was taken on to the next step without further purification.

To a solution of 3-(1-ethyl-1*H*-imidazo[4,5-*c*]pyridin-2-yl)pyrazin-2-amine (1.7 g, 7.08 mmol) in tetrahydrofuran (35 ml) was added *N*-bromosuccinimide (2.27 g, 12.74 mmol) and the reaction mixture was stirred at room temperature for 1 h. Sodium sulfite (aq) (20 ml) was added and this was stirred for 30 min. This was then concentrated until a precipitate formed, which was collected by filtration and washed with water. This afforded 5-bromo-3-(1-ethyl-1*H*-imidazo[4,5-*c*]pyridin-2-yl)pyrazin-2-amine as a yellow solid, which was not further purified (1.76 g, 72% over two steps).

Two drops of water was added to a solution of 5-bromo-3-(1-ethyl-1*H*-imidazo[4,5-*c*]pyridin-2-yl)pyrazin-2-amine (0.80 g, 2.507 mmol), *tert*-butyl (3-oxo-3-((4-(4,4,5,5-tetramethyl-1,3,2-dioxaborolan-2-yl)phenyl)amino)propyl)carbamate (1.03 g, 2.63 mmol), K_2CO_3 (1.04 g, 7.52 mmol) and $PdCl_2$(dppf)-CH_2Cl_2 (0.205 g, 0.251 mmol) in *N*,*N*-dimethylformamide (15 mL) and the reaction mixture was heated to 150ºC for 13 min in a microwave (solution was pre-stirred 8 min). The solution was then partitioned between ethyl acetate (200 ml) and water (200 ml), and the organic phase was washed with further water (2 x 200ml) and then brine (200 ml). It was then dried over Na_2SO_4, filtered and concentrated *in vacuo*. The resulting solid was washed with ethyl acetate to remove the unreacted boronic ester to afford *tert*-butyl (3-((4-(5-amino-6-(1-ethyl-1*H*-imidazo[4,5-*c*]pyridin-2-yl)pyrazin-2-yl)phenyl)amino)-3-oxopropyl)carbamate as a yellow solid (0.50 g, 40 % yield).

tert-Butyl (3-((4-(5-amino-6-(1-ethyl-1*H*-imidazo[4,5-*c*]pyridin-2-yl)pyrazin-2-yl)phenyl)amino)-3-oxopropyl)carbamate (300 mg, 0.597 mmol) was dissolved in dichloromethane (2 mL). To this solution was added trifluoroacetic acid (0.920 mL, 11.94 mmol) and the reaction mixture was stirred at room temperature for 2 h. The solvent was then removed *in vacuo* and the residue was purified by prep-HPLC. This afforded 3-amino-*N*-(4-(5-amino-6-(1-ethyl-1*H*-imidazo[4,5-*c*]pyridin-2-yl)pyrazin-2-yl)phenyl)propanamide (266 mg, 71% yield). 1H NMR (400 MHz, DMSO-d6) δ 10.34 (s, 1H), 9.43 (s, 1H), 8.87 (s, 1H), 8.67 (d, *J* = 6.3 Hz, 1H), 8.28-8.20 (m, 2H), 7.97 (d, *J* = 8.6 Hz, 2H), 7.85-7.74 (m 5H), 5.05 (q, J = 7.0 Hz, 2H), 3.16-3.09 (m, 2H), 2.75 (t, *J* = 6.8 Hz, 3H), 1.52 (t, *J* = 7.0 Hz, 3H). LCMS (Method B) *m/z* 401.3 $[M-H]^-$ (Rt = 0.41 min).

9: 4-(2-(4-Amino-1,2,5-oxadiazol-3-yl)-1-ethyl-7-(piperidin-4-ylmethoxy)-1*H* imidazo[4,5-*c*]pyridin-4-yl)-2-methylbut-3-yn-2-ol

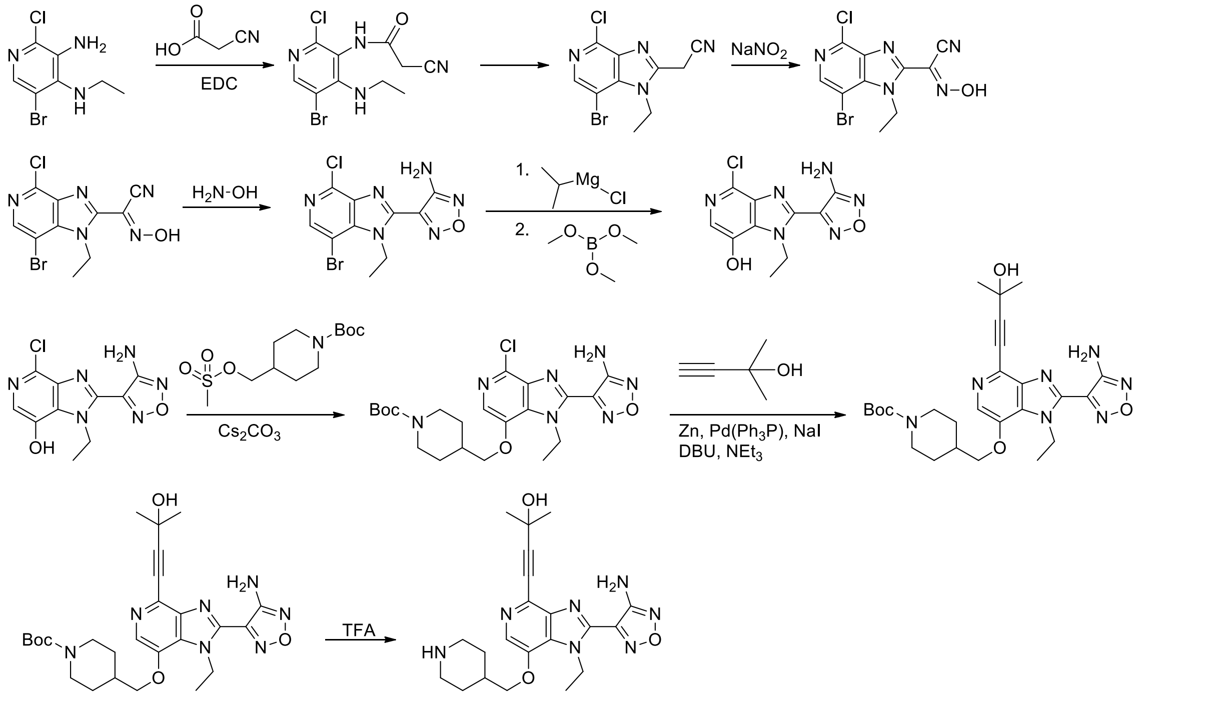

To a solution of 5-bromo-2-chloro-*N*-4-ethylpyridine-3,4-diamine (9.50 g, 37.9 mmol) and 2-cyanoacetic acid (4.84 g, 56.9 mmol) in *N*,*N*-dimethylformamide (100 ml) was added 1-ethyl-3-(3-dimethylaminopropyl)carbodiimide (21.81 g, 114 mmol) followed by *N*-methyl morpholine (20.85 mL, 190 mmol). The reaction mixture was stirred at room temperature for 18 h and was then poured into ethyl acetate (600 ml). This was washed with water (200 ml), brine (150 ml), dried over $MgSO_4$, filtered and evaporated *in vacuo*. The residue was washed with diethyl ether to afford *N*-(5-bromo-2-chloro-4-(ethylamino)pyridin-3-yl)-2-cyanoacetamide as white solid (6.8 g, 57 % yield).

A solution of *N*-(5-bromo-2-chloro-4-(ethylamino)pyridin-3-yl)-2-cyanoacetamide (2.0 g, 6.30 mmol) in 5 mL acetic acid was heated to 90 °C for 1 h. The solvent was removed under reduced pressure to afford 2-(7-bromo-4-chloro-1-ethyl-1*H*-imidazo[4,5-*c*]pyridin-2-yl)acetonitrile) as yellow solid (1.9 g, 100 % yield).

Sodium nitrite (2.418 g, 35.1 mmol) was added portion wise over 20 min to a solution of 2-(7-bromo-4-chloro-1-ethyl-1*H*-imidazo[4,5-c]pyridin-2-yl)acetonitrile (5.0 g,16.69 mmol) in 2N HCl (10 mL) at room temperature. The reaction mixture was stirred at room temperature for a further 3 h and then the resulting precipitate was isolated by filtration. The precipitate was washed with water and dried to afford (*E*)-7-bromo-4-chloro-1-ethyl-*N*-hydroxy-1*H*-imidazo[4,5-c]pyridine-2-carbimidoyl cyanide as yellow solid (4.9 g, 90 % yield).

To a solution of (*E*)-7-bromo-4-chloro-1-ethyl-*N*-hydroxy-1*H*-imidazo[4,5-c]pyridine-2-carbimidoyl cyanide (4.9 g, 14.91 mmol) in 1,4-dioxane (50 mL) was added triethylamine (6.49 g, 64.1 mmol) and hydroxylamine (1.28 g, 38.8 mmol, 55% in water). The reaction mixture was heated to 105 °C for 6 h and then cooled to room

temperature. It was filtered and the filtrate concentrated under reduced pressure to give a brown solid. The solid was suspended in methanol (50 mL) and the suspension heated to 65 °C for 30 min. The solid was collected by filtration and partitioned between water (20 ml) and ethyl acetate (20 mL). The aqueous layer was extracted with further ethyl acetate (2 x 50 mL) and the combined organic layers were dried over Na_2SO_4, filtered and evaporated to afford 4-(7-bromo-4-chloro-1-ethyl-1*H*-imidazo[4,5-*c*]pyridin-2-yl)-1,2,5-oxadiazol-3-amine as yellow solid (2.8 g, 6.03 mmol, 55 % yield).

A solution of 4-(7-bromo-4-chloro-1-ethyl-1*H*-imidazo[4,5-*c*]pyridin-2-yl)-1,2,5-oxadiazol-3-amine (500 mg, 1.46 mmol) in tetrahydrofuran (20 ml) under argon was cooled to -78 °C. To this was added a 2M tetrahydrofuran solution of *iso*propylmagnesium chloride (150 mg, 1.455 mmol) over 20 min. The reaction mixture was stirred for a further 15 min and then trimethyl borate (151 mg, 1.455 mmol) was added dropwise, the temperature inside the flask was kept below -40 °C during addition and then for a further 1h. The reaction mixture was then stirred at room temperature for 3 h and then re-cooled in an ice bath. A 30% aqueous solution of H_2O_2 (10 ml) and of 3M NaOH (aq) (15 ml) were mixed and then added dropwise to the reaction mixture ensuring the reaction mixture remained at 0 °C throughout the addition. After the addition was complete the reaction mixture was stirred at room temperature for 30 min. The reaction mixture was then poured into water (40 ml) and aqueous phase was extracted with ethyl acetate (2 x 50 ml). The combined organic phases were dried over $MgSO_4$, filtered and evaporated to afford 2-(4-amino-1,2,5-oxadiazol-3-yl)-4-chloro-1-ethyl-1*H*-imidazo[4,5-*c*]pyridin-7-ol as pale yellow solid (210 mg, 51 % yield).

To a solution of 2-(4-amino-1,2,5-oxadiazol-3-yl)-4-chloro-1-ethyl-1*H*-imidazo[4,5-*c*]pyridin-7-ol (5.5 g, 19.60 mmol) in *N*,*N*-dimethylformamide (50 mL) was added Cs_2CO_3 (9.58 g, 29.4 mmol). *tert*-Butyl 4-(((methylsulfonyl)oxy)methyl)piperidine-1-carboxylate (8.05 g, 27.4 mmol) was then added and upon complete addition the reaction mixture was heated to 40 °C for 18 h. The reaction mixture was then poured into ethyl acetate (100 ml) and NH_4Cl (aq) (50 ml). The organic phase was washed with water (50 ml), brine (50 ml), dried over Na_2SO_4, filtered and evaporated *in vacuo*. The residue was triturated with *iso*propylalcohol to afford *tert*-butyl 4-(((2-(4-amino-1,2,5-oxadiazol-3-yl)-4-chloro-1-ethyl-1*H*-imidazo[4,5-*c*]pyridin-7-yl)oxy)methyl)piperidine-1-carboxylate as a yellow solid (3.7 g, 40 % yield).

tert-Butyl-4-(((2-(4-amino-1,2,5-oxadiazol-3-yl)-4-chloro-1-ethyl-1*H*-imidazo[4,5-*c*]pyridin-7-yl)oxy)methyl)piperidine-1-carboxylate (1.60 g, 3.35 mmol), 2-methylbut-3-yn-2-ol (1.41 g,16.74 mmol), zinc (0.066 g, 1.00 mmol), sodium iodide (0.151 g, 1.00 mmol), $Pd(Ph_3P)_4$ (0.580 g, 0.50 mmol), triethylamine (1.40 mL, 10.04 mmol) and DBU (1.51 mL, 10.04 mmol) were dissolved in dimethyl sulfoxide (20 mL) in a sealed container under nitrogen. The reaction mixture was heated 85 °C for 6 h. It was then poured into ethyl acetate (50 ml) and the organic phase was washed with water (3 x 30 mL). The organic phase was then dried over Na_2SO_4, filtered and the solvent evaporated *in vacuo*. This afforded *tert*-butyl 4-(((2-(4-amino-1,2,5-oxadiazol-3-yl)-1-ethyl-4-(3-hydroxy-3-methylbut-1-yn-1-yl)-1*H*-imidazo[4,5-*c*]pyridin-7-yl)oxy)methyl)piperidine-1-carboxylate as a mixture which was used in the next step without purification (1.8 g approximately 50 % pure by LCMS).

A solution of *tert*-butyl 4-(((2-(4-amino-1,2,5-oxadiazol-3-yl)-1-ethyl-4-(3-hydroxy-3-methylbut-1-yn-1-yl)-1*H*-imidazo[4,5-*c*]pyridin-7-yl)oxy)methyl)piperidine-1-carboxylate (3.0 g, 5.71 mmol) in dichloromethane (100 mL) was cooled to 0 °C and to this was added trifluoroacetic acid (25 ml). The reaction mixture was stirred at 0 °C for 2 h and then the solvent was removed *in vacuo*. The residue was purified by reverse phase column chromatography (Methanol in water containing 10mmol/L trifluoroacetic acid) to afford 4-(2-(4-amino-1,2,5-oxadiazol-3-yl)-1-ethyl-7-(piperidin-4-ylmethoxy)-1*H*-imidazo[4,5-*c*]pyridin-4-yl)-2-methylbut-3-yn-2-ol, 2 trifluoroacetic acid salt as white solid (1.2 g, 49 % yield). 1H NMR (400 MHz, DMSO-d6) δ 8.63 (bs, 1H), 8.39 (bs, 1H), 8.19 (s, 1H), 7.06 (s, 2H), 4.85 (q, J = 7.0 Hz, 2H), 4.25 (d, J = 5.7 Hz, 2H), 3.37 (d, J = 12.3 Hz, 2H), 2.98 (q, J = 11.9 Hz, 2H), 2.24-2.17 (m, 1H), 1.98 (d, J = 13.5 Hz, 2H), 1.61 (q, J = 11.0 Hz, 2H), 1.54 (s, 6H), 1.46 (t, J = 7.0 Hz, 3H). LCMS (method B) *m/z* 426.3 $[M+H]^+$ (Rt = 0.59 min).

4: 2-((2-((3-(3-Aminopropanamido)phenyl)amino)-5-chloropyrimidin-4-yl)amino)benzamide

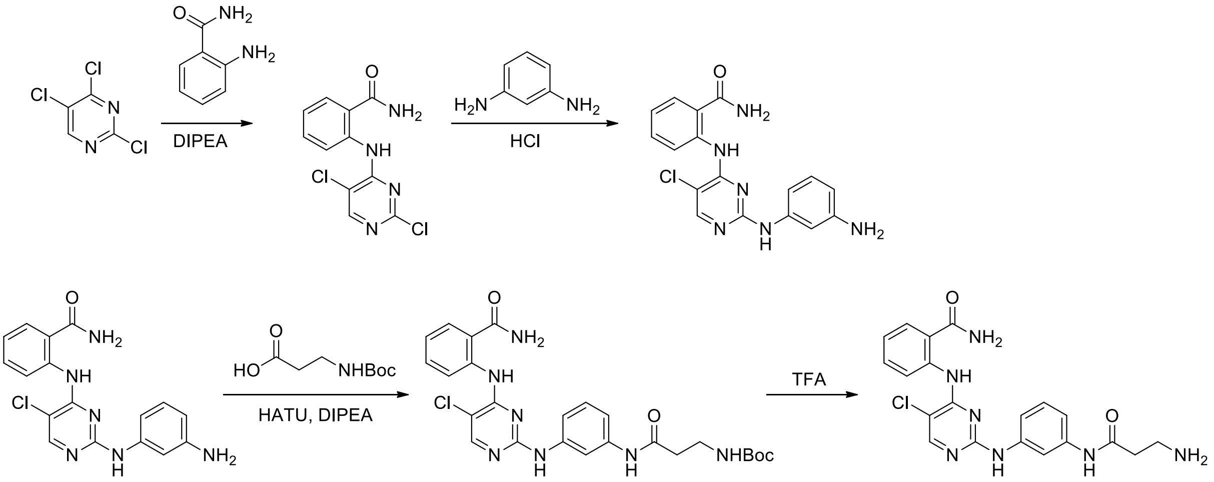

To a solution of 2,4,5-trichloropyrimidine (6.12 g, 33.4 mmol) in *iso*propanol (100 mL) was added 2-aminobenzamide (4.54 g, 33.4 mmol) and DIPEA (6.99 mL, 40.0 mmol). The reaction mixture was stirred at 90 ºC for 18 h and then cooled to room temperature. The precipitate was collected by filtration and washed with *iso*propanol to afford 2-((2,5-dichloropyrimidin-4-yl)amino)benzamide as a colorless solid (8.21 g, 87 % yield).

To a suspension of 2-((2,5-dichloropyrimidin-4-yl)amino)benzamide (5.43 g, 19.19 mmol) and benzene-1,3-diamine (4.23 g, 39.1 mmol) in *iso*propanol (100 ml) was added concentrated HCl (1ml) in a sealed tube. The reaction mixture was heated to 90 ºC for 18 h. After this time as the reaction was not complete, an additional portion of benzene-1,3-diamine (1.0g, 9.28 mmol) and concentrated HCl (1.5 ml) were added to the reaction mixture and it was heated to 90 ºC for a further 18 h. The precipitate was collected by filtration and washed with *iso*propanol to afford 2-((2-((3-aminophenyl)amino)-5-chloropyrimidin-4-yl)amino)benzamide in quantitative yield.

To a solution of 3-((*tert*-butoxycarbonyl)amino)propanoic acid (1.606 g, 8.49 mmol) in *N*,*N*-dimethylformamide (7 mL) was added 2-((2-((3-aminophenyl)amino)-5-

chloropyrimidin-4-yl)amino)benzamide (2.51 g, 7.07 mmol), then HATU (3.23 g, 8.49 mmol) and finally DIPEA (4.94 mL, 28.3 mmol). The reaction mixture was stirred at room temperature for 30 min then another portion of DIPEA (1.236 mL, 7.07 mmol) and HATU (0.807 g, 2.122 mmol) were added and the reaction mixture was stirred for 1h. Finally another portion of 3-((tert-butoxycarbonyl)amino)propanoic acid (0.3 g, 1.58 mmol) was added and the reaction mixture was stirred at room temperature for a further 2 h. It was then poured into water (50 ml) and ethyl acetate (50 mL). A precipitate formed that was suspended in the organic phase, the organic phase was collected, washed with further water (2 x 50 ml) and then filtered. The precipitate was washed with ethyl acetate to afford *tert*-butyl (3-((3-((4-((2-carbamoylphenyl)amino)-5-chloropyrimidin-2-yl)amino)phenyl)amino)-3-oxopropyl)carbamate as a colorless solid (1.50 g, 40 % yield).

To a suspension of *tert*-butyl (3-((3-((4-((2-carbamoylphenyl)amino)-5-chloropyrimidin-2-yl)amino)phenyl)amino)-3-oxopropyl)carbamate (1.9 g, 3.61 mmol) in dichloromethane (20 mL) at room temperature was added trifluoroacetic acid (2 ml). The reaction mixture was stirred at room temperature for 5 h, then diethyl ether was added and the precipitate formed was collected by filtration. The precipitate was washed with further diethyl ether to afford 2-((2-((3-(3-aminopropanamido)phenyl)amino)-5-chloropyrimidin-4-yl)amino)benzamide, trifluoroacetic acid salt as a white solid (1.9 g, 97% yield). 1H NMR (400 MHz, DMSO-d6) δ 12.00 (s, 1H), 10.10 (s, 1H), 9.53 (s, 1H), 8.86 (d, *J* = 8.4 Hz, 1H), 8.31 (s, 1H), 8.22 (s, 1H), 7.84 (s, 1H), 7.84 – 7.64 (m, 4H), 7.45 (t, *J* = 7.9 Hz, 1H), 7.38 (d, *J* = 7.7 Hz, 1H), 7.29 (d, *J* = 8.1 Hz, 1H), 7.23 (t, *J* = 8.0 Hz, 1H), 7.12 (t, *J* = 8.0, 1H), 3.11-3.06 (m, 2H), 2.69 (t, *J* = 6.8 Hz, 2H). LCMS (method B) *m/z* 426.3 $[M+H]^+$ (Rt = 0.41 min)

5: 2-((4-(2-Aminoethoxy)phenyl)amino)-6-(2,6-dichlorophenyl)-8-methylpyrido[2,3-d]pyrimidin-7(8H)-one

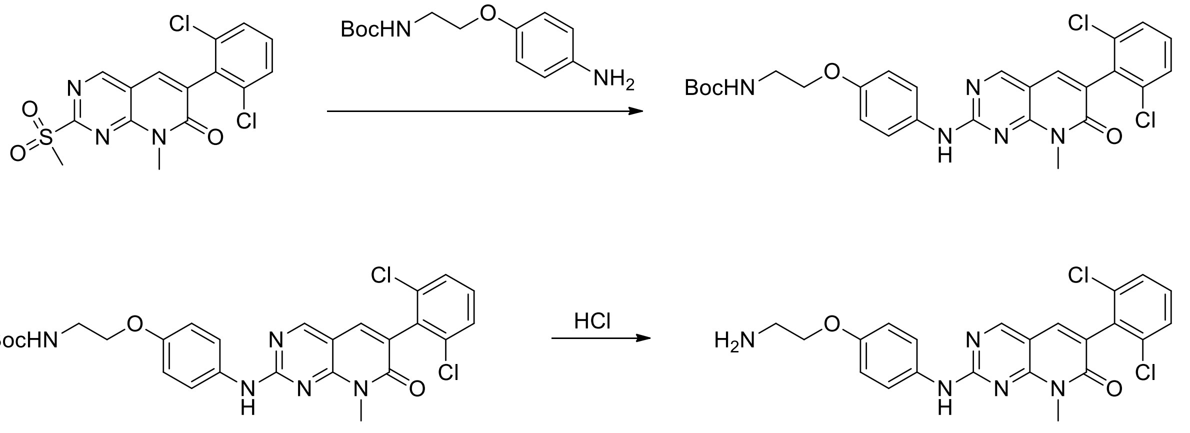

A mixture of 6-(2,6-dichlorophenyl)-8-methyl-2-(methylsulfonyl)pyrido[2,3-*d*]pyrimidin-7(8*H*)-one (1.0 g, 2.60 mmol) and *tert*-butyl 2-(4-aminophenoxy)ethylcarbamate (1.97 g, 7.8 mmol) in diglyme (10 mL) was heated to 140 ºC for 3 hrs. The mixture was cooled to room temperature, diluted with ethylacetate (100 ml) and washed with water (4 x 100 ml). The organic layer was dried over Na_2SO_4, filtered and evaporated. The residue was purified by column chromatography (0-100% ethylacetate in cyclohexane) to afford *tert*-butyl (2-(4-((6-(2,6-dichlorophenyl)-8-

methyl-7-oxo-7,8-dihydropyrido[2,3-*d*]pyrimidin-2-yl)amino)phenoxy)ethyl)carbamate as a yellow solid (828 mg, 57% yield).

To a solution of *tert*-butyl (2-(4-((6-(2,6-dichlorophenyl)-8-methyl-7-oxo-7,8-dihydropyrido[2,3-*d*]pyrimidin-2-yl)amino)phenoxy)ethyl)carbamate (828 mg, 1.49 mmol) in dioxane (5 ml) was added 4.0 M hydrogen chloride solution in dioxane (12 ml) and the reaction mixture was stirred at room temperature for 6 h. The solvent was evaporated and the residue was dissolved in water. The pH of the solution was adjusted to be in the range of 8-9 using a saturated solution of Na_2CO_3 (aq). The resulting precipitate was filtered, washed with water and dried to afford 2-((4-(2-aminoethoxy)phenyl)amino)-6-(2,6-dichlorophenyl)-8-methylpyrido[2,3-*d*]pyrimidin-7(8H)-one as yellow solid (593 mg 87% yield). 1H NMR (400 MHz, DMSO-d6) δ 10.09 (s, 1H), 8.78 (s, 1H), 7.85 (s, 1H), 7.72-7.68 (m, 2H), 7.57 (d, *J* = 8.6, 2H), 7.45 (dd, *J* = 8.8, 7.4, 1H), 6.94 (d, *J* = 8.6 Hz, 2H), 3.89 (t, *J* = 5.8 Hz, 2H), 3.62 (s, 3H), 2.85 (t, *J* = 5.8 Hz, 2H). LCMS (method A) *m/z* 456.2 $[M+H]^+$ (Rt = 5.09 min)

3: *N*-(2-((2-((3-(2-aminoethoxy)phenyl)amino)-5-fluoropyrimidin-4-yl)amino)phenyl)methanesulfonamide

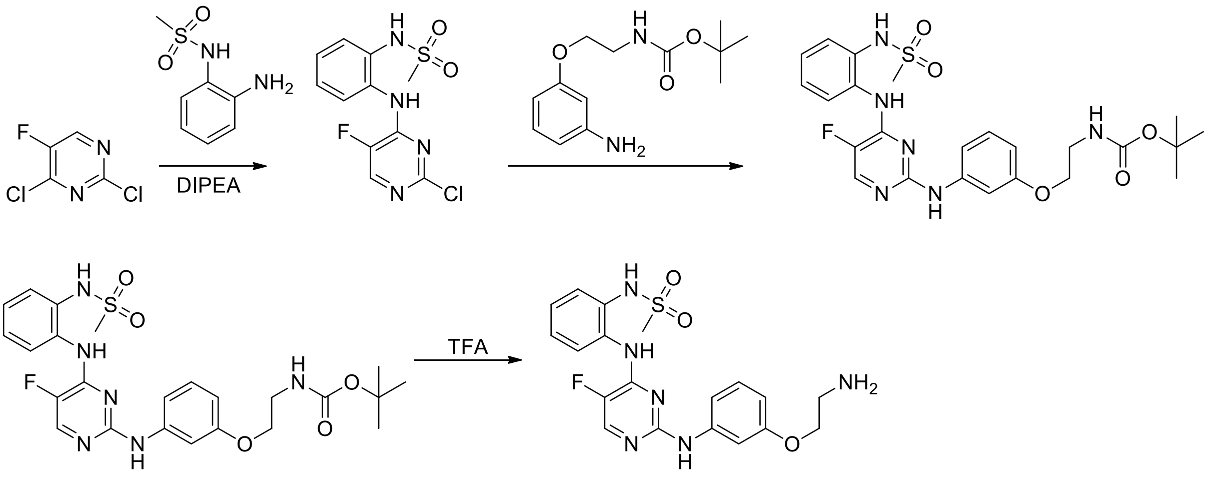

2,4-dichloro-5-fluoropyrimidine (350 mg, 2.10 mmol, 1 equiv) was dissolved in ethanol (6 ml). This solution was cooled to 0 °C and DIPEA (366 ml, 2.10 mmol) was added followed by *N*-(2-aminophenyl)methanesulfonamide (390 mg, 2.10 mmol). The reaction mixture was then slowly warmed to room temperature and stirred for 18 h. The solvent was removed *in vacuo* and the residue was purified by column chromatography (0-100% ethyl acetate in cyclohexane). This afforded *N*-(2((2-chloro-5-fluoropyrimidin-4-yl)amino)phenyl)methanesulfonamide as an orange solid (417 mg, 63% yield).

N-(2((2-chloro-5-fluoropyrimidin-4-yl)amino)phenyl)methanesulfonamide (417 mg, 1.32 mmol) was dissolved in *sec*-butanol (5 ml). To this was added *tert*-butyl (2-(3-aminophenoxy)ethyl)carbamate (365 mg, 1.45 mmol) and the reaction mixture was heated to 100 °C for 18 h. The solvent was removed in vacuo and the residue was purified by column chromatography (0-100% ethyl acetate in cyclohexane). This

afforded *tert*-butyl (2-(3-((5-fluoro-4-((2-(methylsulfonamido)phenyl)amino)pyrimidin-2-yl)amino)phenoxy)ethyl)carbamate as an orange solid (454 mg, 62% yield).

tert-Butyl (2-(3-((5-fluoro-4-((2-(methylsulfonamido)phenyl)amino)pyrimidin-2-yl)amino)phenoxy)ethyl)carbamate (434 mg, 0.815 mmol) was dissolved in dichloromethane (3 ml) and to this was added trifluoroacetic acid (1 ml). The reaction mixture was stirred for 1 h at room temperature. The solvent was removed *in vacuo* and the residue was purified by column chromatography (0-10% methanol in DCM). This afforded *N*-(2-((2-((3-(2-aminoethoxy)phenyl)amino)-5-fluoropyrimidin-4yl)amino)phenyl)methanesulfonamide (205 mg, 58% yield). 1H NMR (400 MHz, DMSO-d6) δ 9.20 (s, 1H), 8.95 (s, 1H), 8.18 (dd, *J* = 7.9, 1.5 Hz, 1H), 8.08 (dd, *J* = 3.5, 1.1 Hz, 1H), 7.42 (s, 1H), 7.27 (d, *J* = 8.3, 1H), 7.23 (d, *J* = 8.3, 1H), 7.10 (dd, *J* = 8.1, 8.1, 1H), 6.96 (dd, *J* = 7.6, 7.6 Hz, 1H), 6.86 (dd, *J* = 8.0, 1H), 6.48 (d, *J* = 8, 1H), 3.94 (t, *J* = 5.4 Hz, 2H), 3.04 (t, *J* = 5.4 Hz, 2H), 2.73 (s, 3H). LCMS (method A) *m/z* 433.2 $[M+H]^+$ (Rt = 3.68 min).

1: *N*-(2-Aminoethyl)-3-((4-(2-(dimethylamino)-4-(3-hydroxyphenyl)thiazol-5-yl)pyrimidin-2-yl)amino)benzamide

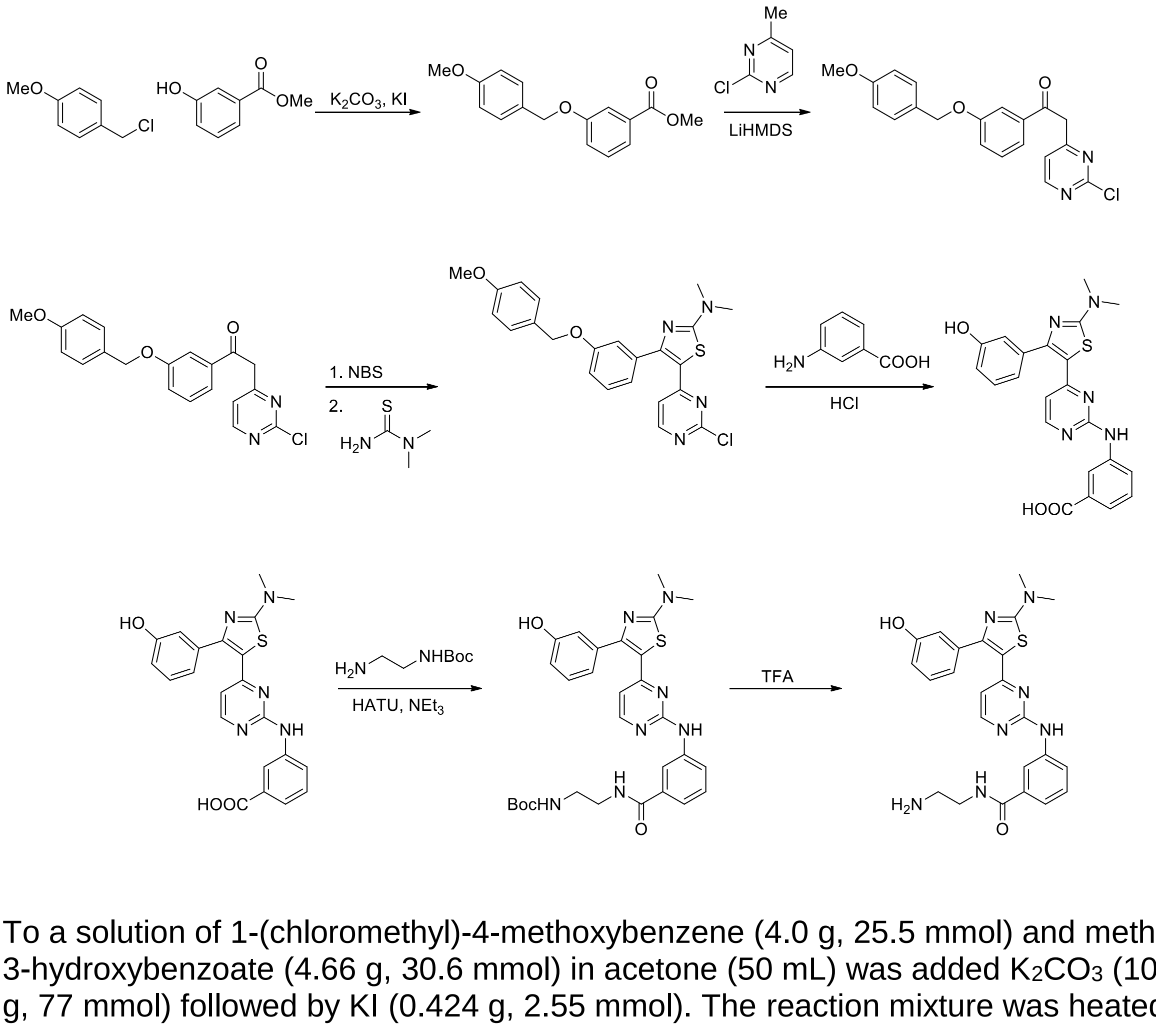

To a solution of 1-(chloromethyl)-4-methoxybenzene (4.0 g, 25.5 mmol) and methyl 3-hydroxybenzoate (4.66 g, 30.6 mmol) in acetone (50 mL) was added K_2CO_3 (10.59 g, 77 mmol) followed by KI (0.424 g, 2.55 mmol). The reaction mixture was heated to reflux for 5 h. Water (50 ml) was added to the cooled reaction mixture and it was extracted with ethyl acetate (3 x 50 mL). The combined organic phases were washed with 1 N NaOH (aq) (2 x 35 mL), brine (50 mL), dried over $MgSO_4$ and filtered. The

solvent was removed to afford methyl 3-((4-methoxybenzyl)oxy)benzoate as a white solid (6.42 g, 93% yield).

Methyl 3-((4-methoxybenzyl)oxy)benzoate (5.0 g, 18.36 mmol) was dissolved in tetrahydrofuran (36 mL) and cooled to 0 °C. LiHMDS (48.2 mL, 48.2 mmol) (1M in tetrahydrofuran) was added in one portion followed by a solution of 2-chloro-4-methylpyrimidine (3.90 g, 30.3 mmol) in tetrahydrofuran (12 ml). The mixture was stirred at 0 °C for 30 min, then warmed to room temperature and stirred for a further 1 h. It was then quenched by addition of saturated NH_4Cl (aq.) (50 mL), and then extracted with ethyl acetate (3 x 50 mL). The combined organic extracts were dried over $MgSO_4$, filtered and the solvent removed *in vacuo*. The residue was dissolved in ethyl acetate (100 mL), and heptane (50 mL) was added, and the precipitate was collected by filtration. This afforded 2-(2-chloropyrimidin-4-yl)-1-(3-((4-methoxybenzyl)oxy)phenyl)ethanone) as a white solid (5.44 g, 80 % yield).

2-(2-chloropyrimidin-4-yl)-1-(3-((4-methoxybenzyl)oxy)phenyl)ethanone) (2.0 g, 5.42 mmol) was dissolved in *N*-*N*-dimethylacetamide (12 ml). To this solution was added *N*-bromosuccinimide (1.01 g, 5.69 mmol) and the reaction mixture was stirred at room temperature for 15 min. 1,1-dimethylthiourea (0.62 g, 5.97 mmol) was then added and the reaction mixture was heated to 60 °C for 1h. It was then poured into water (60 ml) and the precipitate formed was collected by filtration and washed with further water. This solid was then dissolved in dichloromethane and dried over Na_2SO_4, filtered and evaporated. The residue was purified by column chromatography (40% ethyl acetate in heptane) to afford 5-(2-chloropyrimidin-4-yl)-4-(3-((4-methoxybenzyl)oxy)phenyl)-*N*,*N*-dimethylthiazol-2-amine as a yellow solid (1.50 g, 61 % yield).

5-(2-chloropyrimidin-4-yl)-4-(3-((4-methoxybenzyl)oxy)phenyl)-*N*,*N*-dimethylthiazol-2-amine (1.0 g, 2.2 mmol) was dissolved in *iso*propanol (30 ml) and to this was added 3-aminobenzoic acid (0.32 g, 2.3 mmol) and 4M HCl in dioxane (3.31 ml, 13.4 mmol). The reaction mixture was heated to 160 °C in the microwave for 25 min and then poured into ethyl acetate (30 ml). The organic phase was extracted with saturated Na_2CO_3 (aq) (3 x 20 ml). The combined aqueous phases were then acidified to pH 1 with 1M HCl and the precipitate was collected by filtration. This afforded 3-((4-(2-(dimethylamino)-4-(3-hydroxyphenyl)thiazol-5-yl)pyrimidin-2-yl)amino)benzoic acid as a solid (0.825 g, 86 % yield).

3-((4-(2-(dimethylamino)-4-(3-hydroxyphenyl)thiazol-5-yl)pyrimidin-2yl)amino)benzoic acid (0.82 g, 1.88 mmol) was dissolved in *N*,*N*-dimethylformamide (35 ml) and to this was added *tert*-butyl (2-aminoethyl)carbamate (0.33 g, 2.07 mmol) followed by triethylamine (0.52 mL, 3.76 mmol) and finally HATU (0.82 g, 2.16 mmol). The reaction mixture was then stirred at room temperature for 4 h and the solvent removed *in vacuo*. The residue was partitioned between ethyl acetate (20 ml) and 5% $NaHCO_3$ (aq) (20 ml). The aqueous phase was extracted with further ethyl acetate (2 x 20 ml) and the combined organic phases were dried over $MgSO_4$, filtered and evaporated. The residue was triturated with ethyl acetate and filtered to afford *tert*-butyl (2-(3-((4-(2-dimethylamino)-4-(3-hydroxyphenyl)thiazol-5-yl)pyrimidin-2-yl)amino)benzamido)ethyl)carbamate as a yellow solid (685 mg, 63% yield).

tert-Butyl (2-(3-((4-(2-dimethylamino)-4-(3-hydroxyphenyl)thiazol-5-yl)pyrimidin-2-yl)amino)benzamido)ethyl)carbamate (0.745 g, 1.29 mmol) was suspended in

dichloromethane (20 ml) and to this was added trifluoroacetic acid (2.5 ml, 32.4 mmol). This reaction mixture was stirred for 1 h and then the solvent was removed *in vacuo*. This afforded *N*-(2-aminoethyl)-3-((4-(2-(dimethylamino)-4-(3-hydroxyphenyl)thiazol-5-yl)pyrimidin-2-yl)amino)benzamide,trifluoroacetic acid salt as a yellow solid (771 mg, 100% yield). 1H NMR (400 MHz, DMSO-d6) δ 9.68 (s, 1H), 8.52 (t, *J* = 5.6 Hz, 1H), 8.35 (s, 1H), 8.12 (d, J = 5.4 Hz, 1H), 7.90 – 7.74 (m, 3H), 7.44-7.36 (m, 2H), 7.27 (t, *J* = 7.6 Hz, 1H), 6.95 – 6.80 (m, 3H), 6.32 (d, J = 5.4 Hz, 1H), 3.50 (dd, *J* = 6.2, 6.0 Hz, 2H), 3.14 (s, 6H), 2.99 (dd, *J* = 6.2, 6.0 Hz, 2H). LCMS (method B) *m/z* 476.5 $[M+H]^+$ (Rt = 0.49 min).
